# Contrasting cognitive control in the Simon and spatial Stroop tasks regarding their interference with the control of standing balance

**DOI:** 10.1038/s41598-026-56904-9

**Published:** 2026-06-09

**Authors:** Leif Johannsen, Anton Koger, Elisa Ruth Straub, Denise Nadine Stephan, Andrea Kiesel, Iring Koch, Hermann Müller

**Affiliations:** 1 Institute of Psychology, RWTH Aachen University, Jaegerstr. 17/19, 52066 Aachen, Germany; 2 Department of Psychology, University of Freiburg, Freiburg im Breisgau, Germany; 3 Department of Sport Science, University of Giessen, Giessen, Germany

**Keywords:** Balance control, Cognitive conflict resolution, Cognitive-motor interference, Simon task, Spatial Stroop task, Neuroscience, Psychology, Psychology

## Abstract

The scientific understanding of any interaction between cognition and balance control is advanced by methods that capture event-related effects of cognitive processes on balance with high temporal resolution and precision. We developed such an approach to examine how cognitive conflict interferes with the control of body balance during upright standing. Participants stood on a force plate while performing two cognitive conflict paradigms: a Simon task, which according to Kornblum et al.’s dimensional overlap model^41^ mainly induces spatial stimulus–response conflict during response selection, and a Spatial Stroop task, which additionally elicits a stimulus–stimulus conflict during stimulus encoding. By aligning force plate time series data to the onset events of target and response across all trials, we assessed the temporal dynamics of spatial congruency effects on force moment variability as a marker of balance control activity. Across both experimental cognitive tasks, we observed strong congruency effects in cognitive task performance, when considering trials after previous congruent trials. Further, incongruent trials were associated with systematic transient reductions in force moment variability along the mediolateral axis in balance control. These observations are in line with the assumption that the recruitment of cognitive processes for conflict resolution temporarily inhibits, suppresses, or postpones balance adjustments. Importantly, regarding the impact of cognitive interference on body balance, data confirm our previous observations using improved methods and demonstrate that reduction in balance control activity during resolution of cognitive conflict generalizes to a task with multiple conflict loci (Spatial Stroop task). Thereby, this extended range of conflict does not result in correspondingly stronger interference effects in balance control. From a theoretical perspective, the results align with predictive models of postural regulation and intermittent, event-driven accounts of balance control.

**Supplementary Information:**

The online version contains supplementary material available at 10.1038/s41598-026-56904-9.

## Introduction

Maintaining body balance (“balance control”) while quietly standing upright appears motionless but represents a continuously active and dynamic activity. This results from the interplay between gravitational forces on the body and counteracting muscle-generated forces. Stability in standing balance is maintained if the vertical projection of the body’s Centre-of-Mass (CoM) remains within the boundaries of the base of support. The sensorimotor control loop that ensures successful balance control fundamentally requires combining information from various sensory systems that monitor the body’s oscillatory movements relative to the surrounding environment, along with choosing and implementing adequate balance adjustments at the motor level^1–4^.

Balance control during standing is frequently evaluated using posturographic methods that employ force plates to measure body sway through ground-reaction force dynamics^5^. According to Newton’s third law of motion, the ground-reaction force represents the total of forces and moments generated by the neuromuscular system of an individual regulating the angular velocity and acceleration of the body’s Centre-of-Mass as it is affected by gravitational pull, other external and also internal forces acting upon the body^6^. The context-dependent regulation of body oscillation can be examined comprehensively through diverse measures of body motion over a specified time period in addition to measures that capture the complex, longer-term dynamics of body sway^7^.

Cognitive control comprises those executive processes that adapt goal-directed behaviour in dynamic environments that become challenging by changing task demands^8^. The capability to inhibit inappropriate responses and to resolve conflict between competing stimuli is a central aspect. The Simon effect^9^ refers to the interference when there is a stimulus–response (S-R) conflict. In a typical Simon task, participants classify a non-spatial stimulus attribute (e.g., shape, colour, or identity) by pressing a spatially defined response key (e.g., left or right), while the stimulus itself appears at a left or right location that is irrelevant to the task. Responses are faster when the stimulus location corresponds with the response location (congruent) than when it does not (incongruent), even though stimulus location is not part of the instructed task. Presumably, the Simon effect arises because the irrelevant spatial location of the stimulus automatically activates a spatially congruent response tendency^10^. This creates competition between the correct task-relevant response and the automatically activated spatial response. The conflict occurs at the response selection stage when competing motor programmes must be resolved. Similarly, the Spatial Stroop effect^11^ refers to the interference when there is a stimulus–response (S-R) conflict, but in addition there is a stimulus-stimulus (S–S) conflict. In a typical Spatial Stroop task, participants respond to an arrow stimulus that points to the left or the right by pressing the related left or right response key. Thereby, the arrow is either presented at a left or a right location that is task irrelevant. In this setting, the stimulus location is either congruent or incongruent with the response location. Simultaneously, the stimulus location is congruent with the pointing direction of the arrow (e.g. a left pointing arrow presented in the left location), or location and pointing direction are incongruent (e.g. a left pointing arrow presented in the right location). Thus, the Spatial Stroop effect entails stimulus–response as well as stimulus-stimulus conflict. Therefore, the Spatial Stroop task interference is thought to occur during stimulus processing and response selection before response onset^12^.

Two lines of research have been followed to investigate how control of balance and of cognition is mutually linked. First, several recent studies explored whether standing compared to sitting results in interference on the cognitive task using motor requirements as independent variable. Here findings are mixed, while some studies reported some impact of a standing posture (relative to sitting) on performance in diverse cognitive tasks^13–15^, such as the Stroop or the Navon tasks, other studies could not replicate these findings^16–19^. Second, another line of research assessed the impact of a cognitive task on balance control while standing. Consequently, in this second line of research, balance performance becomes the dependent variable, which necessitates an appropriate and sensitive quantification of balance performance. The impact of a cognitive task on balance control while standing can only be determined by assessing how variations in cognitive demands influence balance performance.

Cognitive and sensorimotor processes are known to interfere with one another^20–22^. This is illustrated by balance control as it requires anticipatory planning, multisensory integration to estimate postural state^23^, and the selection of corrective responses that become more reliant on higher-order cognition when aging or pathology slows processing^24–26^. Earlier studies examining how cognition interacts with balance control typically used dual-task paradigms assessing balance with a concurrent cognitive task. When balance remained unaffected but cognitive performance declined, researchers inferred that balance control relies on domain-specific cognitive processes^27,28^. Typically, balance measures are aggregated across a longer period of time and thus across a number of individual trials with the cognitive task.

Only a few studies have tested cognition–balance interference using conflict tasks like the Stroop task. Melzer et al.^29^ showed that a modified Stroop task altered sway in older adults depending on stance width, with reduced sway in narrow stance likely reflecting increased postural stiffness. Similarly, Patterson et al.^30^ reported that older adults performing a spatial Stroop-like task exhibited longer reaction times and less accurate responses in combination with reduced body sway, again suggesting a strategy shift toward postural stiffening. Barra et al.^31^ used auditory verbal and spatial Stroop tasks of varying difficulty together with different stance conditions and observed reduced body sway under dual-tasking, which they interpreted as increased automatization of balance control or postural stiffening.

While these studies following a traditional approach demonstrated that cognitive control and balance control interfere with each other, the approach used cannot pinpoint how specific cognitive processes (such as conflict resolution during stimulus or response processing) affect balance with the precise timing needed for detailed process analysis. Consequently, these studies typically led to broad generalizations about shared cognitive and motor resources rather than specific mechanistic insights. The research examining how attentional control tasks affect standing balance has not produced clear findings regarding the timing of cognitive processes within individual trials. This limitation likely stems from how balance performance is typically measured by averaging time series data over extended periods (tens of seconds to minutes). In contrast, cognitive dual-task research has established that cognitive resource sharing operates at the micro-level (i.e., at the millisecond scale) of specific processing stages during task execution, including response selection processes [see^32,33^, for reviews].

Therefore, we conjecture that investigating the interaction between cognitive control and balance control would benefit from research methods that enable event-related analysis of how cognitive processes influence balance control with high temporal precision. Johannsen et al.^34^ developed an event-related methodology to investigate how cognitive control during cognitive conflict tasks influences balance control during normal upright standing. For this purpose, Johannsen et al.^34^ initially chose the Simon task paradigm as primary focus, because it is widely characterized as involving predominantly stimulus–response (S-R) conflict during response selection. Participants stood on a force plate while performing a Simon task. Instead of investigating the effect of cognitive control on body sway during upright standing estimated over an extended stance period, Johannsen et al.^34^ applied an event-related paradigm, in which the immediate effect of a single cognitive operation in the Simon task on balance control was determined from force moments on a sub-second time scale. Johannsen et al.^34^ aggregated force-plate data in time bins of 150 ms around visual target onset and onset of motor responses (presses of hand-held keys) for examining process-specific interactions between cognitive processes and balance control during standing. First, Johannsen et al.^34^ investigated balance control while performing the Simon task in a target-aligned way, so that differences would reflect predominantly stimulus-based processing conflicts. Second, Johannsen et al.^34^ assessed balance control in a response-aligned way to isolate the effect of cognitive processing preceding overt response production in the Simon task [for similar analysis on EEG distinguishing stimulus- and response-lateralized readiness potential see^35^]. The hypothesis tested was that if cognitive control processes interfered with balance control, we would observe correlates of cognitive conflict in the Simon task in the balance control domain. This previous study showed that resolving response conflict in the Simon task not only produced the expected congruency effect in cognitive performance but also reduced mediolateral sway variability in the temporal period of 150 ms before response onset. This indicates that cognitive conflict resolution processes can momentarily influence balance control in a direction-specific manner. To account for these findings, Johannsen et al.^34^ proposed a “micro-bottleneck” account for control signals for balance control on the one hand and response selection requirements for cognitive tasks on the other hand. This account based on the hypothesis of intermittent balance control signals^36,37^ assuming that balance control operates in intervals of roughly up to two anticipatory balance adjustments per second^38,39^. The authors suggest that this adjustment frequency is linked to physiological constraints, such as neural delays, refractoriness, and neural processing limits. The triggering of corrective events might rely on central processing resources that are also required for response selection in cognitive tasks^40^. Within the framework of the Psychological Refractory Period (PRP), this implies that balance corrections are subject to a central bottleneck: when cognitive processing occupies this stage, the initiation of balance adjustments is temporarily put on hold, resulting in less variable force moment activity in incongruent compared to congruent trials, because the response selection takes longer due to response conflict. Alternatively, however, the interference between cognitive control and balance control might operate also on other cognitive processing stages more in line with general resource-limited assumptions for cognitive and balance control.

### Current study

The present study aimed to test whether the type of cognitive processing conflict, specifically pure stimulus–response (S-R) conflict versus combined stimulus-stimulus (S–S) and S-R conflict, differentially affects the concurrent control of body balance. To this end, we employed a Spatial Stroop task, a conflict paradigm that has not previously been investigated with the event-related force-plate methodology developed by Johannsen et al.^34^. Because the Spatial Stroop task involves both S–S conflict during stimulus encoding and S-R conflict during response selection, it enables us to examine whether the additional locus of conflict produces a qualitatively or quantitatively different pattern of interference with balance control compared to the purely S-R-based Simon task. To provide a direct basis for this comparison, we also included a Simon task that closely followed the procedure of Johannsen et al.^34^, thereby simultaneously replicating the original findings with improved methodology, including temporal jitter between trials and finer temporal resolution (75 ms time bins), and establishing a within-study reference against which the effects of the Spatial Stroop task can be evaluated. The critical question, therefore, is not merely whether cognitive conflict interferes with balance control, but whether the nature and scope of the conflict modulate the magnitude and timing of this interference. To specifically focus on cognitive conflict processing, Johannsen et al.^34^ decided against using the Stroop task, which Kornblum et al.'s^41^ dimensional overlap model indicates involves both perceptual conflict and potentially response conflict as well. Johannsen et al.^34^ instead employed the Simon task as an experimental framework, a paradigm widely recognized for examining cognitive conflict specifically during response selection processes^42,43^. The taxonomy of Kornblum et al.^41^ classified cognitive tasks with respect to the question whether dimensions of stimuli and responses overlap. Overlap within a stimulus set would lead to the potential of stimulus-stimulus conflict, while overlap between stimulus and response sets could lead to stimulus–response conflict. When stimulus and response dimensions overlap, task-irrelevant stimulus information can involuntarily activate a response. However, if the activated response is incorrect, this response tendency based on irrelevant information would require some degree of cognitive control intervention to prevent it from corrupting actual performance. Any differences between the Simon task and the Spatial Stroop task congruency effects lie in the fundamental nature of the conflict being processed according to Kornblum’s dimensional overlap model^44^. In previous studies, two types of conflict were addressed: S–S and S-R conflict [e.g. see review of^45^]. In the current study, therefore, we compared the Simon task with the Spatial Stroop task with the assumption that the Spatial Stroop task would generate even stronger and earlier conflict. We acknowledge that the distinction between the Simon task and the Spatial Stroop task is not as clean-cut as Kornblum’s taxonomy might suggest. For example, Hommel^46,47^ demonstrated that the Simon effect is sensitive to perceptual manipulations, which indicates that multiple processing stages, and not exclusively response selection, may contribute to the effect. Furthermore, the letter stimuli 'T' and 'X' used in our Simon task may carry weak inherent spatial associations (e.g. the location of the ‘X’ button on an Xbox controller), which could introduce a minor stimulus-stimulus component. However, because these letters lack the explicit directional meaning of the arrow stimuli used in the Spatial Stroop task, we consider any such overlap to be minimal relative to the overt spatial S–S conflict present in the Spatial Stroop paradigm. Rather than claiming a strict qualitative dissociation, we therefore use the two tasks as representing different points along a continuum of dimensional overlap, with the Spatial Stroop task incorporating a more pronounced S–S conflict component in addition to the S-R conflict shared by both paradigms. Additionally, the temporal dynamics of the two conflict types might differ between each other. Fischer et al.^48^ reported that the Simon effect typically decays or even reverses when responses are slow, whereas the Spatial Stroop effect may persist or grow over time. If these differing temporal profiles extend to balance control, one might predict that the Spatial Stroop task produces more sustained or temporally extended effects on force moment variability compared to the Simon task.

Based on these considerations, we derived the following specific predictions. First, for both tasks, we expected reduced mediolateral force moment variability in incongruent relative to congruent trials in the response-aligned time series, reflecting interference from S-R conflict resolution with concurrent balance control (replicating Johannsen et al.^34^). Second, because the Spatial Stroop task additionally involves S–S conflict during stimulus encoding, we expected that congruency effects on balance would also be detectable in the target-aligned time series for this task (would argue against an exclusive response selection bottleneck in favour of a more generalised account). Third, owing to its dual conflict sources, the Spatial Stroop task might produce temporally more extended or larger congruency effects on balance than the Simon task. The absence of such task differences would suggest that balance interference is driven primarily by S-R conflict processes, irrespective of the presence of additional S–S conflict. Finally, we explored whether the temporal profile of balance interference differed between the two tasks.

## Methods

### Participants

Ninety healthy young adults (M = 21.4 years, SD = 2.7; female = 67, male = 23; right-handed = 81) were recruited for the current study in two laboratories with basically identical setup in Aachen (n = 72) and in Freiburg (n = 18). Participants were naïve regarding the hypotheses of the experiment, reported normal or corrected-to-normal vision and had neither neurological, musculoskeletal, psychiatric, nor any other relevant medical diagnoses nor did they show any current balance impairments. Based on Johannsen et al.^34^, where a t-test for dependent measures found a within-subject effect size of congruency in the Simon task in the mediolateral body sway direction of dz = 0.41, a sample size of 80 participants was calculated for an actual power of 0.95^49^. Ten additional participants were recruited to follow a conservative approach. All participants were informed about the study protocol and gave signed written informed consent. Ethical approval was obtained prior to the study from the local faculty’s ethical committee at the RWTH Aachen University (2022_013_FB7_RWTH Aachen) and the study protocol was conducted in accordance with the ethical research standards of the amended declaration of Helsinki.

### Equipment and cognitive tasks

The experiment was conducted in a dimly lit, quiet laboratory. A 31.5 inch LCD monitor with a refresh rate of 144 Hz and a resolution of 3840 × 2160 pixels was used for the presentations of visual targets in both cognitive tasks described below. The monitor was placed in front of the participants at about 85 cm distance and a height of 130 cm. Targets were the white-on-black letters T and X (both 1 cm width and 1.5 cm height) and left- or right-pointing arrows (both 1.8 cm width and 0.8 cm height) that were presented 16 cm left or right from a fixation cross (1.5 cm width and height, presented horizontally and vertically centralized). The input device for the manual responses was a game controller (Rii PC controller USB gamepad; dimensions: 16 × 11.5 × 7 cm; weight: 161 g). Participants were instructed to press left and right keys on the controller using their left and right index fingers. Participants held the game controller with both hands in front of their upper body and they were told to hold the controller relaxed and rather close to their trunk with the elbows close to both sides of the body and the arms slightly flexed upwards at the elbow joints so that the hands were level with the height of their navel. They did not receive explicit instruction to stand as quietly as possible but were instructed to prevent any voluntary balance disturbance by moving body limbs or shifting weight only.

Body sway was recorded using a multi-component force plate type Kistler 9260AA with a temporal resolution of 1000 Hz. The force plate uses piezoelectric 3-component force sensors to register the forces and moments exerted on the plate in the three spatial directions. Sway recordings were performed on a second computer using the BioWare software (version 5.3.0.7; Kistler Group, 2012). The computer running the cognitive tasks communicated via a parallel port interface with the force plate computer, where additional channels were registered as analog input devices sampled also at 1 kHz and synchronized with the force plate data acquisition. These channels recorded uniquely encoded trigger signals sent at several trial events (e.g. fixation onset, target onset, and response onset) in each trial for later processing of behavioural data and force plate time series data^50^.

**Fig. 1:**
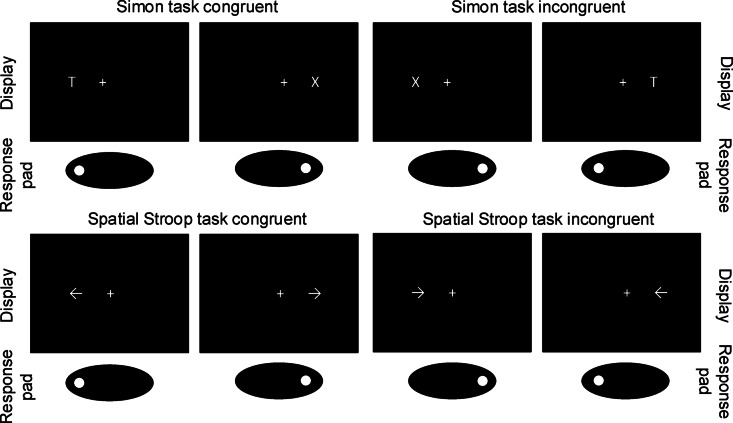
Target-response configurations of a Simon task (top row) and a Spatial Stroop task (bottom row). Participants reacted with a button press on a game controller according to the identity of the letter target or the pointing direction of an arrow irrespective of the location.

While standing, participants performed the two cognitive tasks (Fig. 1) within a single session (duration 90 min to 2 h). The order of both tasks was counter-balanced across participants to control for any systematic order effects. At the beginning of each part of the session, participants performed at least ten practice trials to familiarize themselves to the experimental procedure of each task. In each task, participant performed ten blocks of roughly 280 s duration of continuous standing. They were offered breaks between the individual blocks. Each of the ten blocks contained 80 trials so that 800 trials were presented in total for each task.

The cognitive tasks were run on a computer using PsychoPy [version 2023.2.3;^51^]. A Simon task was modelled after the version used in Johannsen et al.^34^. It comprised a visual 2-alternative forced choice task, in which the letters ‘T’ and ‘X’ were presented either on the left or the right of a central fixation. Fixation and target were presented in white on a black background. The letter ‘T’ required participants to respond with a left button press, and the letter ‘X’ asked for a right button response while ignoring the presentation location. A match between the required manual response and the side of the target resulted in a congruent trial, while a mismatch between instructed target response and target location defined an incongruent trial.

A Spatial Stroop task [modelled after^52^] was designed in analogy to the Simon task with arrows pointing to the left or right that were presented on the left or right side of the fixation. Participants were required to respond to the direction in which an arrow was pointing (left button press when an arrow pointed to the left and right button press when the arrow pointed to the right) while ignoring the presentation location. Again, a match between the required manual response and the side of the target resulted in a congruent trial, while a mismatch between instructed target response and target location defined an incongruent trial. In both cognitive tasks, the sequence of congruent and incongruent trials within a block was random.

Following a fixation cross of 200 ms duration, a target was presented horizontally to the left or the right of the fixation cross. The target and fixation cross remained visible until a manual response occurred, or a period of 1.5 s had passed without a response. The reaction time (RT) period was followed by 350 ms of feedback in case of an incorrect response (a correct response resulted in a blank screen) and each trial ended with a 200 ms blank screen inter-trial interval. Each intertrial interval was further extended by a blank screen before the onset of the next fixation with randomly jittered durations in the range from 500 to 2500 ms. This jitter period ensured that participants were unable to anticipate the exact onset of the next trial based on any rhythmic structure in the trial procedure. In addition, the fixation onset jittering decoupled slow oscillations in body sway from trial timing when aggregating over any individual trials. Hence, the maximum response-target interval was 3250 ms (minimum of 1250 ms). During data analysis, we considered only trials with RTs shorter than 1500 ms, that is, trials with overall durations shorter than 4750 ms.

### Post‑processing of force plate data and parameter extraction

Time series data post-processing and parameter extraction were performed in MATLAB 2023b (The Mathworks, Natick, MA) as well as RStudio (version 2025.05.0 + 496; R version 4.4.2) using custom-prepared algorithms. Forces and moments of the force plate were used to characterize the control of body sway. A dual-pass moving average with sliding window width of 19 ms was applied for general smoothing of all time series data to remove any high frequency signals, artefacts or by-products, which are not directly associated with supraspinal control of sway, such as electromagnetic noise or peripheral and spinal reactions (muscle twitches or spinal reflexes). The experimental blocks for each cognitive task were segmented into individual trials using the trial event signals that indicated the onset of fixation.

Single trials were excluded from post-processing if the entire duration of a trial surpassed 4750 ms, for example responses were not recorded in the 1500 ms interval after target onset. These trials were labelled as “unresponsive” and were not considered for further processing. Additionally, trials with incorrect responses, correct trials following an incorrect response, and the very first trials of each block were excluded. In the Simon task, a total proportion of 7.5% of all trials were excluded from data analysis, of which 3.6% were excluded as response errors, 3.4% as correct responses following an error, and 0.5% as correct responses with RTs longer than 1500 ms. In the Spatial Stroop task, a total of 6.1% of all trials were excluded from data analysis, of which 3.0% were excluded as response errors, 2.9% as correct responses following an error, and 0.2% as correct responses with RTs longer than 1500 ms.

Like in Johannsen et al.^34^, two major branches of data processing were pursued by aligning all the time series data to the time points of the onset of the target (target-aligned) as well as the onset of the manual response (response-aligned). Around the anteroposterior (AP) and mediolateral (ML) axis for a specified time bin, the current state of balance was analysed as the standard deviation (SD) of the ground reaction force moment (units in Nmm). Variability of the force moment within a time bin expresses the amount of activity applied by the neuromuscular system for the control of standing balance. Greater variability would indicate balance adjustments being more likely to demonstrate their effect within this short period. We chose a temporal bin width of 75 ms (amounts to 75 datapoint at 1 kHz sample rate) duration for improved time course resolution compared to Johannsen et al.^34^. An increased number of temporal bins compared to our precursor study allowed us to describe the moment variability before and after each trial event in more detail. For target-aligned trial time series, therefore, six temporal bins were extracted from 150 ms before until 300 ms after target onset. Six temporal bins were also extracted for the response-aligned trial time series from 300 ms before to 150 ms after response onset.

### Design

Performance in the Simon task and the Spatial Stroop task, in terms of RT and error proportions (EP), needed to be assessed to validate that a robust effect of cognitive conflict was induced by the respective target stimuli. For both cognitive tasks, congruency of the current trial and congruency of the previous trial were used as the independent variables. We considered previous trial congruence as additional independent variable, because ample of research [see also our own previous study,^34^, e.g.^53^] has shown that the impact of congruency in the current trial is larger after a previously congruent rather than incongruent trial. (Several accounts have been proposed for explaining this so-called 'congruence sequence effect’ (e.g. Botvinick et al.,^58^; Dignath et al.,^59^; Egner,^60^; Hommel et al.,^56^). To investigate the impact of the current trial’s congruency manipulation on balance control, it is sufficient for us to consider trials with previously congruent trials only.) Potential factors such as laterality of the target stimulus presentation and laterality of the manual reactions were not considered of interest due to lack of any a priori hypotheses because any specific effect of laterality would be cancelled out by calculating averages across sides.

The dependent variables were RT and EP. RTs were directly measured by the presentation software and validated by signals of stimulus and response triggers in the force plate data. The dependent variables were based on response timing information acquired through the target stimulus presentation software directly as well as by the trial event trigger signals sent to the data acquisition board of the force plate setup. With respect to replicating our previous study^34^, we restricted our analysis of the force-plate data to trials that followed a congruent previous trial because we expected the strongest effects of cognitive conflict in these trials [see also^54^ for discussion]. As dependent variables representing balance control in both the AP and ML directions of sway, we calculated the standard deviation of the force moment time series within the respective time bins of each single trial of a participant.

## Results

All statistical computations were performed in R Studio (2025.09.1 Build 401; R version 4.4.2). For the force moment parameters, statistical analyses were performed for the target-aligned and the response-aligned time series data. The variability of the moments acting around the anteroposterior and mediolateral axes was analysed in terms of its standard deviation within each 75 ms time bin. Force moment variability was natural log-transformed before statistical analysis. This transformation was applied to reduce the positive skew that is characteristic of standard deviation measures and to stabilize variance across conditions, thereby better satisfying the assumptions of normality and homogeneity of variance required for parametric analyses of variance.

To avoid contradiction between inferential system^55^, we selected the commonly used significance testing approach for evaluating effects and interactions were evaluated at an alpha level of p < 0.05.

## Analysis of manual reactions

### Comparison between both cognitive tasks

To look at the effects of conflict in the two cognitive tasks, RT and EP were analysed with congruency as within-subject factor. Previous trial congruency was added as a second within-subject factor to assess specific sequential conflict adaptation effects, and cognitive task (Simon vs. Spatial Stroop) as a third within-subject factor of the repeated-measures analyses of variance (ANOVAs). Tables 1 and 2 provide the descriptive statistics of the reaction times and error proportions for both cognitive tasks combined and individually and Table 3 summarizes the inferential test statistics of ANOVAs for the manual reaction times and the error proportions.

**Table 1:** Descriptive statistics of the reaction times for both cognitive tasks as a function of congruency and previous trial congruency.

	Cognitive conflict task											
	Both tasks included				Simon task				Spatial Stroop task			
	Previous trial congruency				Previous trial congruency				Previous trial congruency			
Current trial congruency	Congruent		Incongruent		Congruent		Incongruent		Congruent		Incongruent	
	M	SD	M	SD	M	SD	M	SD	M	SD	M	SD
Incongruent	527	59	495	60	529	63	498	64	525	55	492	56
Congruent	458	57	502	61	468	60	508	70	448	52	495	51
Congruency effect	69	27	− 7	26	61	22	− 10	26	77	28	− 3	26

M: mean, S: standard deviation.

**Table 2:** Descriptive statistics of the error proportions for both cognitive tasks as a function of congruency and previous trial congruency.

	Cognitive conflict task											
	Both tasks included				Simon task				Spatial Stroop task			
	Previous trial congruency				Previous trial congruency				Previous trial congruency			
Current trial congruency	Congruent		Incongruent		Congruent		Incongruent		Congruent		Incongruent	
	M	SD	M	SD	M	SD	M	SD	M	SD	M	SD
Incongruent	7.1	4.9	2.3	2.3	7.1	5.1	2.4	2.4	7.1	4.7	2.2	2.1
Congruent	0.9	1.2	2.9	3.1	1.2	1.4	3.7	3.6	0.6	1.0	2.1	2.2
Congruency effect	6.3	4.6	− 0.6	2.7	5.9	4.7	− 1.3	3.0	6.5	4.5	0.1	2.1

M: mean, S: standard deviation.

**Table 3:** Test statistics of ANOVAs for manual reaction times and error proportions with task condition as within-subject factor (rows 1 and 2) and for each individual cognitive task (Simon task: rows 3 and 4; Spatial Stroop task: rows 5 and 6).

		F, p, partial eta^2						
		Main effect	Main effect	Main effect	Interaction	Interaction	Interaction	Interaction
		Task condition	Current trial congruency	Previous trial congruency	Task condition by current trial congruency	Task condition by previous trial congruency	Current trial congruency by previous trial congruency	Task condition by current trial congruency by previous trial congruency
Both tasks included	Reaction times	**F(1, 89) = 4.58, p = .04, η**_**p**_^**2**^** = 0.05**	**F(1, 89) = 286.98, *p* < 0.001, η**_**p**_^**2**^** =0.76**	**F(1, 89) = 60.79, *p* < 0.001, η**_**p**_^**2**^** = 0.41**	**F(1, 89) = 20.65, *p* < 0.001, η**_**p**_^**2**^** = 0.19**	F(1, 89) = 1.98, p = 0.16, **η**_**p**_^**2**^ = 0.02	**F(1, 89) = 790.18, *p* < 0.001, η**_**p**_^**2**^** = 0.90**	**F(1, 89) = 10.95, p = 0.001, η**_**p**_^**2**^** = 0.11**
	Error proportion	**F(1, 89) = 9.84, p = 0.002, η**_**p**_^**2**^** = 0.10**	**F(1, 89) = 128.42, *p* < 0.001, η**_**p**_^**2**^** = 0.59**	**F(1, 89) = 103.71, *p* < 0.001, η**_**p**_^**2**^** = 0.54**	**F(1, 89) = 12.92, *p* < 0.001, η**_**p**_^**2**^** = 0.13**	**F(1, 89) = 7.50, p = 0.007, η**_**p**_^**2**^** = 0.08**	**F(1, 89) = 197.46, *p* < 0.001, η**_**p**_^**2**^** = 0.69**	**F(1, 89) = 4.34, p = 0.04, η**_**p**_^**2**^** = 0.05**
Simon task	Reaction times		**F(1, 89) = 193.28, *p* < 0.001, η**_**p**_^**2**^** = 0.68**	**F(1, 89) = 17.62, *p* < 0.001, η**_**p**_^**2**^** = 0.17**			**F(1, 89) = 414.90, *p* < 0.001, η**_**p**_^**2**^** = 0.82**	
	Error proportion		**F(1, 89) = 59.05, *p* < 0.001, η**_**p**_^**2**^** = 0.40**	**F(1, 89) = 37.61, *p* < 0.001, η**_**p**_^**2**^** = 0.30**			**F(1, 89) = 166.97, *p* < 0.001, η**_**p**_^**2**^** = 0.65**	
Spatial stroop task	Reaction times		**F(1, 89) = 214.39, *p* < 0.001, η**_**p**_^**2**^** = 0.71**	**F(1, 89) = 57.00, *p* < 0.001, η**_**p**_^**2**^** = 0.39**			**F(1, 89) = 866.01, *p* < 0.001, η**_**p**_^**2**^** = 0.91**	
	Error proportion		**F(1, 89) = 148.79, *p* < 0.001, η**_**p**_^**2**^** = 0.63**	**F(1, 89) = 95.57, *p* < 0.001, η**_**p**_^**2**^** = 0.52**			**F(1, 89) = 167.13, *p* < 0.001, η**_**p**_^**2**^** = 0.65**	

Significant effects or interactions are indicated in bold.

**Fig. 2:**
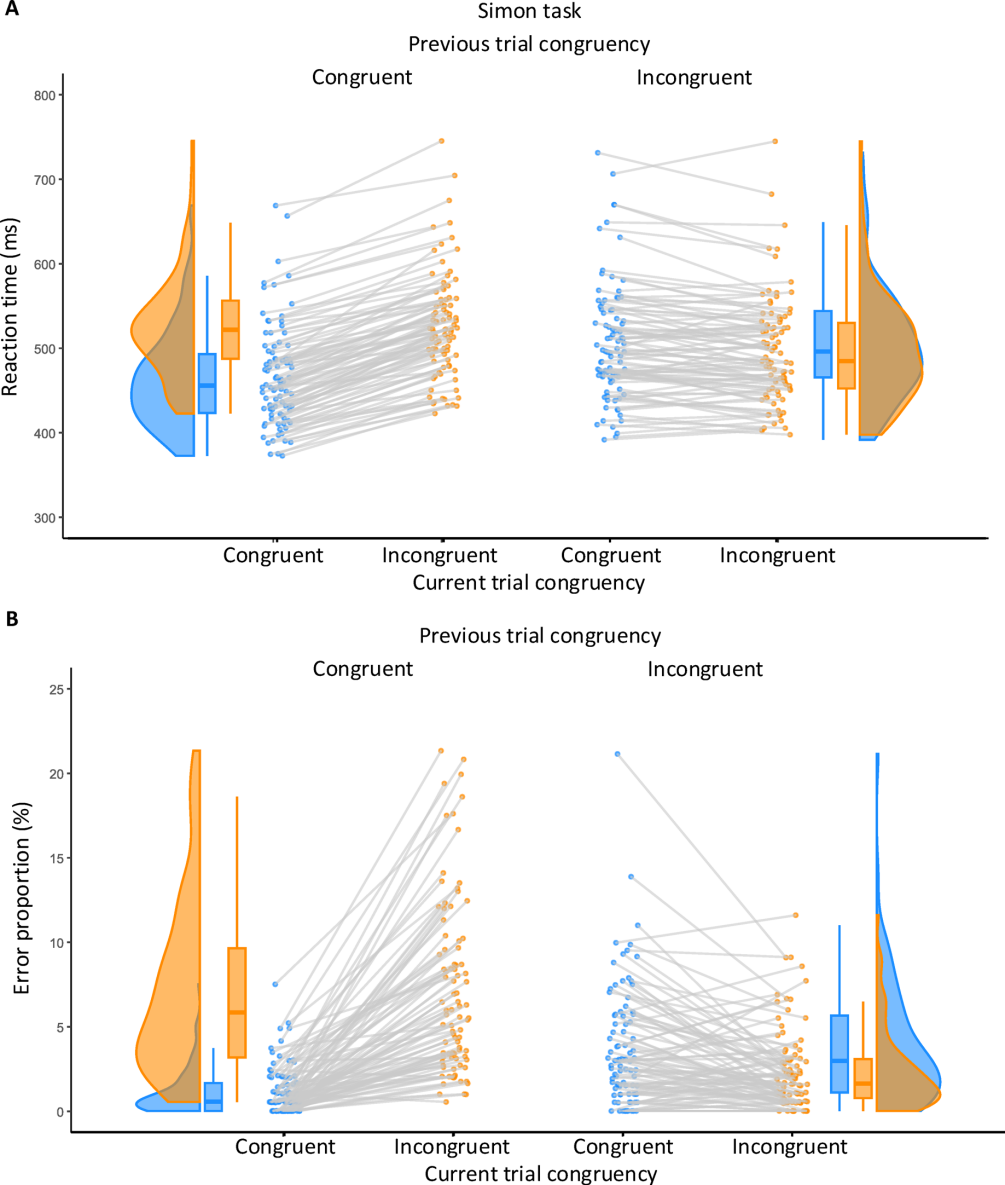
Raincloud plots of the (**A**) reaction times and (**B**) error proportions in the Simon task as a function of the congruency of the current trial and of the previous trial.

**Fig. 3:**
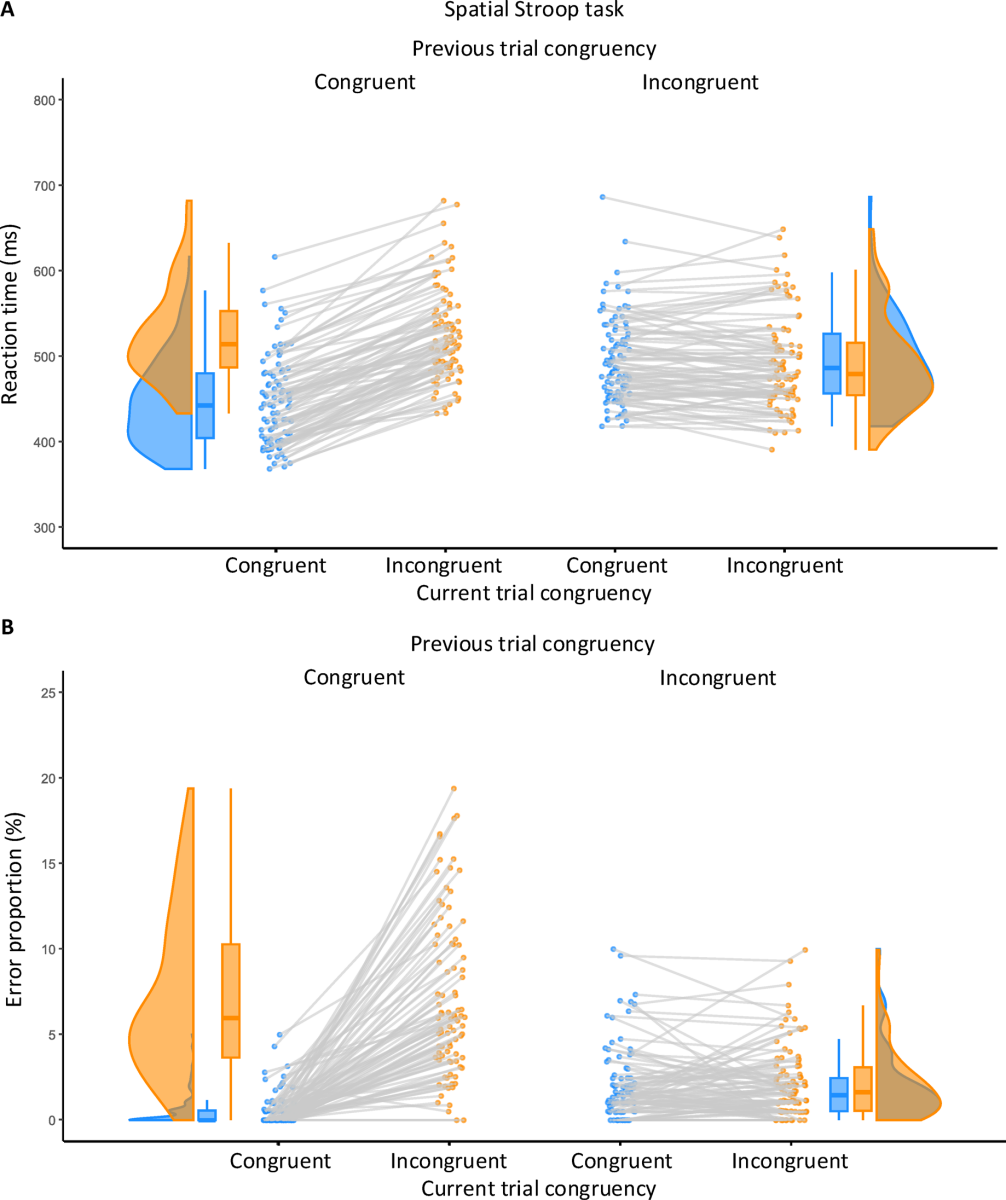
Raincloud plots of the (**A**) reaction times and (**B**) error proportions in the Spatial Stroop task as a function of the congruency of the current trial and of the previous trial.

Both cognitive tasks produced robust congruency effects. Figures 2 and 3 present the average reaction times and error proportions as raincloud plots for both tasks as a function of current and previous trial congruency. We computed an ANOVA with the factor congruence of the current trial, congruence in of the previous trial and cognitive task (Simon task vs. Spatial Stroop task) for both RTs and error rates. Across both tasks, incongruent trials were associated with longer RTs (M = 511 ms, SD = 61) and higher error proportions (M = 4.7%, SD = 4.5) than congruent trials (RT: M = 480 ms, SD = 63; EP: M = 1.9%, SD = 2.6; both ps < 0.001). Strong congruence sequence effects were observed: after congruent trials (RT: 69 ms; EP: 6.3%), whereas after incongruent trials, the congruency effect was eliminated and even slightly reversed (RT: − 7 ms; EP: − 0.6%; all interactions *p* < 0.001).

The Spatial Stroop task produced overall shorter RTs (M = 490 ms, SD = 60) than the Simon task (M = 501 ms, SD = 68; p = 0.04), fewer errors (Spatial Stroop task : M = 3.0%, SD = 3.8; Simon task: M = 3.6%, SD = 4.1; p = 0.002), and a somewhat larger congruency effect (RT: 36 ms vs. 26 ms; EP: 3.4% vs. 2.1%; cognitive task by congruency interaction ps < 0.001). In addition, the three-way interaction between cognitive task, congruency, and previous-trial congruency was significant in both RT and EP (ps ≤ 0.04). This latter interaction demonstrates a difference regarding the sequential modulation of the congruence effects for both cognitive tasks: whereas the Simon task showed a reversed congruency effect after incongruent trials (RT: − 10 ms, p < 0.001; EP: − 1.3%, *p* < 0.001), the Spatial Stroop task showed a complete elimination of the congruency effect after incongruent trials without reversal (RT: 3 ms, p = 0.16; EP: 0.1%, p = 0.52). Individual analyses for each cognitive task are reported in the supplementary materials.

### Analysis of force moment variability

The following description of statistical results concerns only those trials, where the previous trial was congruent, because for these trials the effects of congruency are larger compared to for trials after incongruent trials. We analysed force moment variability separately in the AP and ML directions for both target-aligned and response-aligned times series. As will be reported below, the effect of cognitive task was not significant and did not interact with congruency or time bin. Therefore, analyses of balance performance for each of the two cognitive tasks separately are reported in the supplementary materials.

### Response-aligned time series analyses of congruency effects in both cognitive tasks

Tables 4 and 5 list the descriptive statistics of the response-aligned and target-aligned force moment variability for both cognitive tasks as well as the Simon task and the Spatial Stroop task and each congruency condition and time bin. Table 6 contains the test statistics of the ANOVAs of the congruency effect for the target-aligned and response-aligned moment variability for both cognitive tasks combined and the individual tasks. The main effects and interactions of cognitive task, congruency and the progression across the time bins were assessed as within-subject factors of a repeated-measures analyses of variance (ANOVA).

**Table 4:** Descriptive statistics of the log-transformed anteroposterior force moment variability for both cognitive tasks as a function of congruency and time bin.

Target-aligned	Cognitive conflict task	Time bin	− 150 to − 75 ms		− 75 ms to target onset		target onset to 75 ms		75 to 150 ms		150 to 225 ms		225 to 300 ms	
		Congruency	M	SD	M	SD	M	SD	M	SD	M	SD	M	SD
	Both tasks included	Incongruent	− 2.33	0.52	− 2.32	0.52	− 2.33	0.53	− 2.33	0.52	− 2.35	0.52	− 2.39	0.51
		Congruent	− 2.33	0.51	− 2.32	0.51	− 2.32	0.52	− 2.33	0.52	− 2.35	0.52	− 2.39	0.51
		Congruency effect	0.006	0.11	0.004	0.11	− 0.004	0.11	0.002	0.10	0.002	0.10	0.005	0.10
	Simon task	Incongruent	− 2.34	0.52	− 2.32	0.51	− 2.33	0.52	− 2.34	0.52	− 2.36	0.53	− 2.39	0.51
		Congruent	− 2.34	0.51	− 2.32	0.50	− 2.33	0.51	− 2.33	0.51	− 2.36	0.51	− 2.40	0.52
		Congruency effect	− 0.008	0.11	− 0.003	0.11	− 0.006	0.10	− 0.007	0.10	− 0.004	0.10	0.008	0.10
	Spatial stroop task	Incongruent	− 2.31	0.53	− 2.31	0.53	− 2.32	0.53	− 2.32	0.52	− 2.34	0.52	− 2.39	0.50
		Congruent	− 2.33	0.51	− 2.32	0.53	− 2.32	0.53	− 2.33	0.53	− 2.35	0.53	− 2.39	0.51
		Congruency effect	0.021	0.11	0.012	0.11	− 0.003	0.11	0.012	0.10	0.007	0.10	0.002	0.10

Response-aligned	Cognitive conflict task	Time bin	− 300 to − 225 ms		− 225 to − 150 ms		− 150 to − 75 ms		− 75 ms to response onset		Response onset to 75 ms		75 to 150 ms	
		Congruency	M	SD	M	SD	M	SD	M	SD	M	SD	M	SD
	Both tasks included	Incongruent	− 2.38	0.51	− 2.38	0.50	− 2.40	0.49	− 2.40	0.49	− 2.37	0.48	− 2.37	0.49
		Congruent	− 2.37	0.51	− 2.37	0.51	− 2.39	0.51	− 2.38	0.50	− 2.35	0.49	− 2.36	0.49
		Congruency effect	− 0.018	0.11	− 0.012	0.10	− 0.005	0.10	− 0.018	0.10	− 0.017	0.09	− 0.007	0.11
	Simon task	Incongruent	− 2.39	0.51	− 2.38	0.50	− 2.41	0.49	− 2.41	0.49	− 2.38	0.48	− 2.38	0.50
		Congruent	− 2.37	0.51	− 2.37	0.51	− 2.40	0.51	− 2.39	0.50	− 2.36	0.49	− 2.36	0.49
		Congruency effect	− 0.019	0.11	− 0.015	0.11	− 0.003	0.10	− 0.016	0.10	− 0.025	0.09	− 0.020	0.12
	Spatial Stroop task	Incongruent	− 2.38	0.51	− 2.38	0.51	− 2.39	0.50	− 2.40	0.49	− 2.35	0.48	− 2.35	0.49
		Congruent	− 2.36	0.51	− 2.37	0.52	− 2.38	0.51	− 2.38	0.50	− 2.35	0.49	− 2.36	0.49
		Congruency effect	− 0.018	0.11	− 0.009	0.10	− 0.008	0.10	− 0.020	0.10	− 0.009	0.09	0.008	0.10

M: mean, S: standard deviation.

**Table 5:** Descriptive statistics of the log-transformed mediolateral force moment variability for both cognitive tasks as a function of congruency and time bin.

Target-aligned	Cognitive conflict task	Time bin	− 150 to − 75 ms		− 75 ms to target onset		target onset to 75 ms		75 to 150 ms		150 to 225 ms		225 to 300 ms	
		Congruency	M	SD	M	SD	M	SD	M	SD	M	SD	M	SD
	Both tasks included	Incongruent	− 2.60	0.52	− 2.60	0.53	− 2.62	0.53	− 2.62	0.52	− 2.65	0.53	− 2.69	0.50
		Congruent	− 2.59	0.53	− 2.58	0.54	− 2.60	0.54	− 2.63	0.54	− 2.65	0.53	− 2.68	0.50
		Congruency effect	− 0.008	0.12	− 0.02	0.12	− 0.02	0.12	0.003	0.11	− 0.003	0.11	− 0.006	0.11
	Simon task	Incongruent	− 2.60	0.50	− 2.60	0.50	− 2.63	0.50	− 2.63	0.50	− 2.66	0.50	− 2.69	0.47
		Congruent	− 2.59	0.51	− 2.57	0.53	− 2.60	0.53	− 2.63	0.53	− 2.65	0.50	− 2.69	0.49
		Congruency effect	− 0.013	0.11	− 0.029	0.12	− 0.035	0.12	− 0.003	0.13	− 0.003	0.13	− 0.008	0.11
	Spatial Stroop task	Incongruent	− 2.59	0.55	− 2.59	0.56	− 2.61	0.56	− 2.61	0.55	− 2.64	0.55	− 2.68	0.54
		Congruent	− 2.59	0.54	− 2.58	0.56	− 2.60	0.55	− 2.62	0.55	− 2.64	0.55	− 2.67	0.52
		Congruency effect	− 0.003	0.12	− 0.012	0.11	− 0.005	0.11	0.010	0.09	− 0.003	0.09	− 0.004	0.10

Response-aligned	Cognitive conflict task	Time bin	− 300 to − 225 ms		− 225 to − 150 ms		− 150 to − 75 ms		− 75 ms to response onset		Response onset to 75 ms		75 to 150 ms	
		Congruency	M	SD	M	SD	M	SD	M	SD	M	SD	M	SD
	Both tasks included	Incongruent	− 2.68	0.50	− 2.69	0.51	− 2.73	0.50	− 2.72	0.48	− 2.69	0.49	− 2.69	0.46
		Congruent	− 2.65	0.51	− 2.66	0.52	− 2.69	0.51	− 2.69	0.50	− 2.68	0.50	− 2.67	0.47
		Congruency effect	− 0.024	0.11	− 0.029	0.12	− 0.036	0.12	− 0.031	0.10	− 0.017	0.12	− 0.011	0.12
	Simon task	Incongruent	− 2.68	0.47	− 2.70	0.48	− 2.73	0.48	− 2.73	0.45	− 2.71	0.45	− 2.70	0.43
		Congruent	− 2.66	0.49	− 2.67	0.50	− 2.69	0.50	− 2.70	0.48	− 2.69	0.48	− 2.68	0.46
		Congruency effect	− 0.021	0.13	− 0.030	0.11	− 0.036	0.11	− 0.025	0.11	− 0.017	0.13	− 0.012	0.13
	Spatial Stroop task	Incongruent	− 2.67	0.53	− 2.69	0.53	− 2.72	0.52	− 2.72	0.51	− 2.68	0.52	− 2.68	0.50
		Congruent	− 2.64	0.53	− 2.66	0.55	− 2.68	0.53	− 2.68	0.52	− 2.67	0.52	− 2.67	0.49
		Congruency effect	− 0.028	0.10	− 0.029	0.13	− 0.037	0.13	− 0.038	0.10	− 0.016	0.11	− 0.010	0.11

M: mean, S: standard deviation.

**Table 6:** Test statistics of ANOVAs of the congruency effect for the moment variability in each temporal bin (target-aligned and response-aligned) for the both cognitive tasks in both directions of body sway.

		F, p, partial eta^2						
		Temporal bin	− 150 to − 75 ms	− 75 ms to target onset	target onset to 75 ms	75 to 150 ms	150 to 225 ms	225 to 300 ms
Target− aligned	Both cognitive tasks	Anteroposterior	F(1, 89) = 0.50, p = 0.48, **η**_**p**_^**2**^ < 0.01	F(1, 89) = 0.29, p = 0.59, **η**_**p**_^**2**^ < 0.01	F(1, 89) = 0.32, p = 0.57, **η**_**p**_^**2**^ < 0.01	F(1, 89) = 0.14, p = 0.71, **η**_**p**_^**2**^ < 0.01	F(1, 89) = 0.05, p = 0.82, **η**_**p**_^**2**^ < 0.01	F(1, 89) = 0.48, p = 0.49, **η**_**p**_^**2**^ < 0.01
		Mediolateral	F(1, 89) = 0.69, p = 0.41, **η**_**p**_^**2**^ < 0.01	**F(1, 89) = 5.24, p = 0.02, η**_**p**_^**2**^** = 0.06**	**F(1, 89) = 5.47, p = 0.02, η**_**p**_^**2**^** = 0.06**	F(1, 89) = 0.17, p = 0.679, **η**_**p**_^**2**^ < 0.01	F(1, 89) = 0.20, p = 0.656, **η**_**p**_^**2**^ < 0.01	F(1, 89) = 0.62, p = 0.435, **η**_**p**_^**2**^ < 0.01
	Simon task	Anteroposterior	F(1, 89) = 0.51, p = 0.48, **η**_**p**_^**2**^ < 0.01	F(1, 89) = 0.08, p = 0.77, **η**_**p**_^**2**^ < 0.01	F(1, 89) = 0.26, p = 0.61, **η**_**p**_^**2**^ < 0.01	F(1, 89) = 0.46, p = 0.50, **η**_**p**_^**2**^ < 0.01	F(1, 89) = 0.12, p = 0.73, **η**_**p**_^**2**^ < 0.01	F(1, 89) = 0.58, p = 0.45, **η**_**p**_^**2**^ < 0.01
		Mediolateral	F(1, 89) = 1.21, p = 0.27, **η**_**p**_^**2**^ = 0.01	**F(1, 89) = 5.31, p = 0.02, η**_**p**_^**2**^** = 0.06**	**F(1, 89) = 7.68, p = 0.007, η**_**p**_^**2**^** = 0.08**	F(1, 89) = 0.06, p = 0.81, **η**_**p**_^**2**^ < 0.0	F(1, 89) = 0.05, p = 0.82, **η**_**p**_^**2**^ < 0.01	F(1, 89) = 0.44, p = 0.51, **η**_**p**_^**2**^ < 0.01
	Spatial Stroop task	Anteroposterior	F(1, 89) = 2.98, p = 0.09,** η**_**p**_^**2**^ = 0.03	F(1, 89) = 1.00, p = 0.32,** η**_**p**_^**2**^ = 0.01	F(1, 89) = 0.07, p = 0.79,** η**_**p**_^**2**^ < 0.01	F(1, 89) = 1.45, p = 0.23,** η**_**p**_^**2**^ = 0.02	F(1, 89) = 0.44, p = 0.51,** η**_**p**_^**2**^ < 0.01	F(1, 89) = 0.03, p = 0.87,** η**_**p**_^**2**^ < 0.01
		Mediolateral	F(1, 89) = 0.05, p = 0.83,** η**_**p**_^**2**^ < 0.01	F(1, 89) = 1.01, p = 0.32,** η**_**p**_^**2**^ = 0.01	F(1, 89) = 0.17, p = 0.68, **η**_**p**_^**2**^ < 0.01	F(1, 89) = 0.99, p = 0.32,** η**_**p**_^**2**^ = 0.01	F(1, 89) = 0.09, p = 0.76,** η**_**p**_^**2**^ < 0.01	F(1, 89) = 0.12, p = 0.73,** η**_**p**_^**2**^ < 0.01
	Temporal bin	− 300 to − 225 ms	− 225 to − 150 ms	− 150 to − 75 ms	− 75 ms to response onset	response onset to 75 ms	75 to 150 ms	
Response-aligned	Both cognitive tasks	Anteroposterior	**F(1, 89) = 6.28, p = 0.01, η**_**p**_^**2**^** = 0.07**	F(1, 89) = 2.72, p = 0.10, **η**_**p**_^**2**^ = 0.03	F(1, 89) = 0.46, p = 0.50, **η**_**p**_^**2**^ < 0.01	**F(1, 89) = 5.54, p = 0.021, η**_**p**_^**2**^** = 0.06**	**F(1, 89) = 7.04, p = 0.009, η**_**p**_^**2**^** = 0.07**	F(1, 89) = 0.73, p = 0.395, **η**_**p**_^**2**^ < 0.01
		Mediolateral	**F(1, 89) = 7.72, p = 0.007, η**_**p**_^**2**^** = 0.08**	**F(1, 89) = 10.85, p = 0.001, η**_**p**_^**2**^** = 0.11**	**F(1, 89) = 14.68, p < 0.001, η**_**p**_^**2**^** = 0.14**	**F(1, 89) = 14.30, *p* < 0.001, η**_**p**_^**2**^** = 0.14**	F(1, 89) = 3.12, p = 0.08, η_p_^2^ = 0.03	F(1, 89) = 1.63, p = 0.20, **η**_**p**_^**2**^ = 0.02
	Simon task	Anteroposterior	F(1, 89) = 2.60, p = 0.11,** η**_**p**_^**2**^ = 0.03	F(1, 89) = 1.57, p = 0.21,** η**_**p**_^**2**^ = 0.02	F(1, 89) = 0.08, p = 0.79,** η**_**p**_^**2**^ < 0.01	F(1, 89) = 2.39, p = 0.13,** η**_**p**_^**2**^ = 0.03	**F(1, 89) = 6.91, p = 0.01, η**_**p**_^**2**^ = 0.07 *	F(1, 89) = 2.90, p = 0.09,** η**_**p**_^**2**^ = 0.03
		Mediolateral	F(1, 89) = 2.42, p = 0.12,** η**_**p**_^**2**^ = 0.03	**F(1, 89) = 6.28, p = 0.014, η**_**p**_^**2**^** = 0.07**	**F(1, 89) = 9.96, p = 0.002, η**_**p**_^**2**^** = 0.10**	**F(1, 89) = 5.00, p = 0.03, η**_**p**_^**2**^** = 0.05**	F(1, 89) = 1.65, p = 0.20,** η**_**p**_^**2**^ = 0.02	F(1, 89) = 0.80, p = 0.37,** η**_**p**_^**2**^ < 0.01
	Spatial Stroop task	Anteroposterior	F(1, 89) = 2.50, p = 0.12, **η**_**p**_^**2**^ = 0.03	F(1, 89) = 0.82, p = 0.37, **η**_**p**_^**2**^ < 0.01	F(1, 89) = 0.54, p = 0.46, **η**_**p**_^**2**^ < 0.01	F(1, 89) = 3.86, p = 0.05, **η**_**p**_^**2**^ = 0.04	F(1, 89) = 0.88, p = 0.35, **η**_**p**_^**2**^ < 0.01	F(1, 89) = 0.56, p = 0.46, **η**_**p**_^**2**^ < 0.01
		Mediolateral	**F(1, 89) = 7.14, p = 0.009, η**_**p**_^**2**^** = 0.07**	**F(1, 89) = 4.72, p = 0.03, η**_**p**_^**2**^** = 0.05**	**F(1, 89) = 6.93, p = 0.01, η**_**p**_^**2**^** = 0.07**	**F(1, 89) = 11.97, *p* < 0.001, η**_**p**_^**2**^** = 0.12**	F(1, 89) = 1.77, p = 0.19, **η**_**p**_^**2**^ = 0.02	F(1, 89) = 0.80, p = 0.37, **η**_**p**_^**2**^ < 0.01

Significant effects or interactions are indicated in bold.

**Fig. 4:**
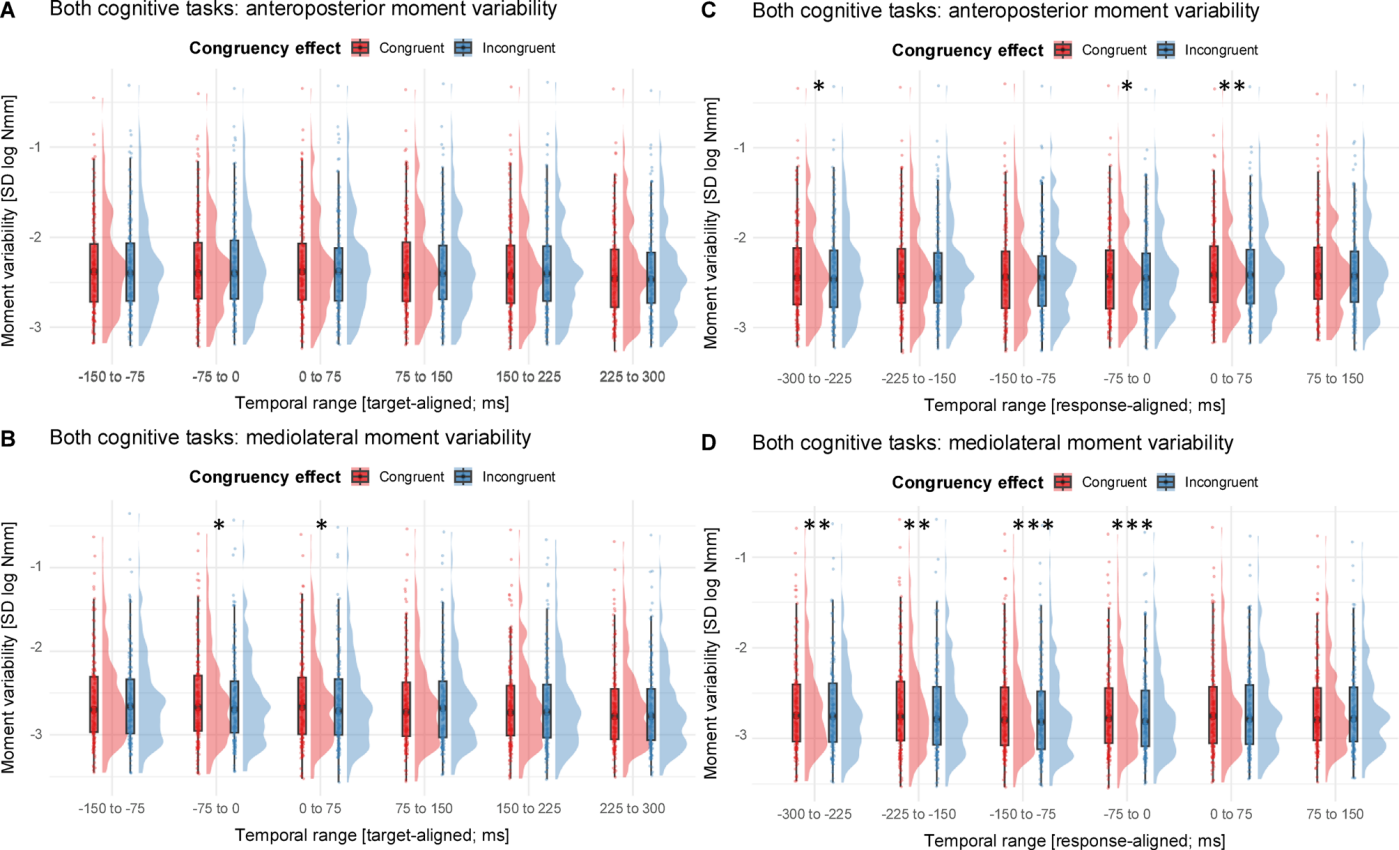
Raincloud plots of the distributions of the anteroposterior (**A**) and mediolateral (**B**) log-transformed standard-deviation of moment variability in both cognitive tasks across the target-aligned temporal bins as a function of congruency of the current trial (only for trials where the previous trial was congruent too) for each extracted time bin (75 ms width; from 150 ms before to 300 ms after target onset). Distributions of the log-transformed standard-deviation of moment variability across the response-aligned temporal bins in the anteroposterior (**C**) and mediolateral (**D**: from 300 ms before to 150 ms after response onset).

For the response-aligned force moment time series, AP moment variability demonstrated significant main effects for congruency (F(1, 89) = 6.07, MSE = 0.0149, p = 0.016, η_p_^2^ = 0.06) and change across time bins (F(2.31, 205.65) = 11.85, MSE = 0.0148, *p* < 0.001, ƞ_p_^2^ = 0.12; Fig. 4b) only, but no interaction between both factors. Moment variability was lower in incongruent trials (M = − 2.38, SD 0.49) than congruent trials (M = -2.37, SD 0.50). Across the time bins, moment variability reduced until the time bin from 75 ms to response onset and then began to rise again (M_1_ = − 2.37, SD_1_ 0.51; M_2_ = − 2.37, SD_2_ 0.51; M_3_ = − 2.39, SD_3_ 0.50; M_4_ = − 2.39, SD_4_ 0.49; M_5_ = − 2.36, SD_5_ 0.49; M_6_ = − 2.36, SD_6_ 0.49). The cognitive task had no specific influence on moment variability or its time course.

For the ML moment variability when aligned to response-onset, significant main effects were observed for congruency (F(1, 89) = 11.98, MSE = 0.0280, *p* < 0.001, ƞ_p_^2^ = 0.12) and time bins (F(2.22, 197.56) = 14.47, MSE = 0.0164, *p* < 0.001, ƞ_p_^2^ = 0.14), in addition to an interaction between both factors (F(3.63, 322.98) = 2.63, MSE = 0.0042, p = 0.040, ƞ_p_^2^ = 0.03; Fig. 4d). Moment variability was lower in incongruent trials (M = − 2.70, SD 0.49) than congruent trials (M = -2.67, SD 0.50). Across the time bins, moment variability reduced until the time bin from 75 ms to response onset and then began to rise again (M_1_ = − 2.66, SD_1_ 0.504; M_2_ = -2.68, SD_2_ 0.513; M_3_ = − 2.71, SD_3_ 0.50; M_4_ = − 2.71, SD_4_ 0.490; M_5_ = − 2.69, SD_5_ 0.492; M_6_ = − 2.68, SD_6_ 0.466). The interaction between congruency and time bins resulted from only the four time bins before response-onset showing reduced moment variability in incongruent trials (M_1_ = − 2.68, SD_1_ 0.499; M_2_ = − 2.69, SD_2_ 0.506; M_3_ = − 2.73, SD_3_ 0.498; M_4_ = − 2.72, SD_4_ 0.480) compared to congruent trials (M_1_ = − 2.65, SD_1_ 0.510; M_2_ = − 2.66, SD_2_ 0.521; M_3_ = − 2.69, SD_3_ 0.514; M_4_ = − 2.69, SD_4_ 0.499; Fig. 4d). The cognitive task had no specific influence on moment variability or its time course as a three-way interaction between cognitive task, congruency and time bin was not observed (F(3.84, 341.76) = 0.29, MSE = 0.0033, p = 0.878, ƞ_p_^2^ < 0.01). Likewise, a comparison between both cognitive tasks regarding their maximum reduction of force moment variability during incongruent trials did not demonstrate a difference in their maximum congruency effect sizes (F(1, 89) = 0.02, MSE = 0.0101, p = 0.899, ƞ_p_^2^ < 0.01).

### Target-aligned time series analyses of congruency effects in both cognitive tasks

For the target-aligned AP force moment time series, a change across the six time bins was observed (F(2.94, 261.79) = 71.42, MSE = 0.0067, *p* < 0.001, ƞ_p_^2^ = 0.45; Fig. 4a) only. This was caused by a general reduction in moment variability, independent of congruency, that became observable in the time bin from 150 to 225 ms after target onset (M_1_ = -2.33, SD_1_ 0.51; M_2_ = − 2.32, SD_2_ 0.52; M_3_ = -2.32, SD_3_ 0.52; M_4_ = -2.33, SD_4_ 0.52; M_5_ = − 2.35, SD_5_ 0.52; M_6_ = − 2.39, SD_6_ 0.51). A similar change across time bins was also seen in the ML direction (F(2.85, 254.02) = 98.61, MSE = 0.0085, *p* < 0.001, ƞ_p_^2^ = 0.53) but with an earlier start of reduction observable at the time bin from target onset to 75 ms after (M_1_ = − 2.59, SD_1_ 0.52; M_2_ = -2.59, SD_2_ 0.54; M_3_ = − 2.61, SD_3_ 0.53; M_4_ = − 2.62, SD_4_ 0.53; M_5_ = − 2.65, SD_5_ 0.53; M_6_ = − 2.68, SD_6_ 0.50). In addition, an interaction between change across time bins and the congruency condition occurred (F(3.47, 308.88) = 2.58, MSE = 0.0044, p = 0.045, ƞ_p_^2^ = 0.03). The time bins from 75 ms before to target onset (F(1, 89) = 5.24, MSE = 0.0035, p = 0.024, ƞ_p_^2^ = 0.06) and from target onset to 75 ms after target onset (F(1, 89) = 5.47, MSE = 0.0035, p = 0.022, ƞ_p_^2^ = 0.06) demonstrated reduced moment variability in incongruent trials (M_2_ = − 2.60, SD_2_ 0.53; M_3_ = − 2.62, SD_3_ 0.53) compared to congruent trials (M_2_ = − 2.58, SD_2_ 0.54; M_3_ = − 2.60, SD_3_ 0.54; Fig. 4c).

### Sensitivity analysis: force moment variability including all trials of both cognitive tasks

Our primary analyses of balance data were restricted to trials following a congruent previous trial, because the congruency effect in cognitive task performance is substantially larger under this condition, thereby maximizing sensitivity for detecting interference with balance control. However, this restriction excludes approximately half of the available data, which raises questions about the generalizability of the findings [see^56^, regarding potential feature-integration confounds in sequential-trial selection]. Therefore, we performed additional analyses where we included all trials irrespective of previous trial congruency and where we included only trials with previously incongruent trials.

### Anteroposterior force moment variability

The analysis of response-aligned AP force moment variability including all trials revealed only a main effect of time bin (F(2.01, 178.80) = 15.77, MSE = 0.029, *p* < 0.001, ηp^2^ = 0.15) reflecting the general modulation of moment variability across the time bins that was also observed in the primary analysis. The main effect of congruency did not reach significance (F(1, 89) = 3.03, MSE = 0.014, p = 0.085, ηp^2^ = 0.03), and congruency did not interact with previous-trial congruency, time bin, or cognitive task (all ps > 0.093). The four-way interaction between cognitive task, congruency, previous-trial congruency, and time bin likewise failed to reach significance (F(3.47, 309.01) = 2.04, MSE = 0.0041, p = 0.098, ηp^2^ = 0.02). When only trial where the previous trial was incongruent were included, the effect of time bin (F(2.29, 203.98) = 14.15, *p* < 0.001, ηp^2^ = 0.14) was significant only. These results confirm the primary analysis for AP moment variability that cognitive conflict did not systematically modulate balance control along the AP axis, irrespective of whether the analysis was restricted to trials following a congruent, incongruent trial or included the full dataset.

### Mediolateral force moment variability

The analysis of response-aligned ML force moment variability including all trials revealed a significant main effect of congruency (F(1, 89) = 6.22, MSE = 0.025, p = 0.014, ηp^2^ = 0.07) with reduced moment variability in incongruent compared to congruent trials. A main effect of time bin was also present (F(2.12, 188.60) = 17.97, MSE = 0.026, *p* < 0.001, ηp^2^ = 0.17). The congruency effect was modulated by previous-trial congruency (F(1, 89) = 6.01, MSE = 0.030, p = 0.016, ηp^2^ = 0.06), which is consistent with a congruence sequence effect. Also, a congruency by time bin interaction was observed (F(3.49, 310.19) = 2.70, MSE = 0.0042, p = 0.038, ηp^2^ = 0.03) indicating that the congruency effect on ML moment variability varied across the response-aligned time bins. Neither the two-way interaction between cognitive task and congruency (F(1, 89) = 0.86, MSE = 0.023, p = 0.357, ηp^2^ < 0.01) nor the three-way interaction between cognitive task, congruency, and time bin (F(3.38, 300.94) = 0.30, MSE = 0.0043, p = 0.847, ηp^2^ < 0.01) reached significance. Thus, the Simon and the Spatial Stroop task did not differ in their magnitude or temporal progression of their congruency-related effects on balance control. Additional significant interactions between cognitive task and time bin (F(2.86, 254.82) = 3.83, MSE = 0.0071, p = 0.012, ηp^2^ = 0.04) and between cognitive task, previous-trial congruency, and time bin (F(3.80, 338.23) = 3.28, MSE = 0.0035, p = 0.013, ηp^2^ = 0.04) indicated task-specific differences in the temporal modulation of moment variability that were unrelated to the congruency manipulation. When only trial where the previous trial was incongruent were included, effects of time bin (F(2.57, 229.11) = 14.14, MSE = 0.013, *p* < 0.001, ηp^2^ = 0.14) and a cognitive task by time bin interaction (F(3.54, 314.92) = 6.67, MSE = 0.0048, *p* < 0.001, ηp^2^ = 0.07) were found.

## Discussion

To elucidate the mechanisms behind interference between cognitive processes and the control of body balance, we pursued a novel event-related methodology. We examined whether two types of spatial cognitive conflict, combined stimulus-stimulus (S–S) and stimulus–response (S-R) conflict in a Spatial Stroop task and stimulus–response (S-R) conflict in a Simon task, differentially interfere with the concurrent control of body balance^34,57^. Both tasks produced robust cognitive congruency effects and, critically, both were accompanied by transient reductions in mediolateral force moment variability during incongruent relative to congruent trials. The time range in which congruency influenced moment variability before response onset (e.g. in terms of an earlier onset of a difference between congruent and incongruent trials in the Spatial Stroop task) did not differ qualitatively between the two cognitive tasks (no three-way interaction between cognitive task, congruency, and temporal bin).

### Strength and robustness of the cognitive conflict

Both tasks showed robust congruence sequence effects, where the congruency effect was modulated by the previous trial’s congruency. After congruent trials, participants displayed the standard congruency effects in both the Simon and the Spatial Stroop tasks. After incongruent trials, congruency effects were eliminated in both tasks, with the Simon task additionally showing a reversal of the congruency effect [for similar findings see e.g.^10^]. This sequential modulation is consistent with patterns widely reported in the literature, although its underlying mechanisms remain debated. Accounts range from conflict-driven cognitive control adjustments^58^ to feature-integration and repetition-based explanations^56^, strategic and affective factors^59^, and multi-level learning^60^. Notably, the reversal observed in the Simon task has been argued to make interpretation in terms of genuine cognitive adaptation more difficult^61^, as it may partly reflect repetition or strategic processes. A detailed discussion of these mechanisms is beyond the scope of this study; we included previous-trial congruency primarily as a factor to identify the trials with the largest congruency effects (e.g. those following congruent trials), thereby maximising sensitivity for detecting interference with balance control.

A sensitivity analysis including all trials confirmed the primary analysis: cognitive conflict produced a transient reduction in mediolateral force moment variability, and this effect does not differ qualitatively between the Simon and the Spatial Stroop tasks. The magnitude of the main effect of congruency was attenuated relative to the primary, post-congruent-only analysis (ηp^2^ = 0.07 in the full analysis against ηp^2^ = 0.12 in the primary analysis). The analysis that included only trials where the previous trial was incongruent indicated no effects of congruency or interactions between congruency and time bin (both ηp^2^ < 0.01). Congruency effects are known to be substantially reduced or reversed following incongruent trials. Nonetheless, the main effect of congruency on response-aligned ML moment variability remained significant, and the interaction between congruency and time bin indicated that the temporal dynamics of the effect were preserved. In contrast, anteroposterior moment variability was unaffected by congruency regardless of trial selection, paralleling the primary analysis. These findings indicate that the reported balance interference effect is not an artefact of the restriction to post-congruent trials, and they address concerns that removing half of the data might have inflated the apparent sensitivity of our method.

### Temporal localisation of the interference effects on balance

Kornblum’s taxonomy presumes that both the Simon and the Spatial Stroop tasks involve S-R conflict during response selection. Our previous study^34^ detected reduced ML moment variability within a single 150 ms time bin before response onset. The present study improved temporal resolution by halving time-bin width to 75 ms, extended the analysis window from 300 ms before to 150 ms after response onset, and introduced temporal jitter to eliminate predictability of trial onsets. Under these improved conditions, the strongest congruency effect on moment variability was detected from 150 to 75 ms before response onset, with weaker effects in the two neighbouring time bins, suggesting that the underlying cognitive process may be active across roughly 225 ms prior to the response. Provided that balance control is subject to neuromuscular delays and the body’s inertia, the impact of any critical cognitive process may precede any effects observable in the force plate data.

Although Kornblum’s taxonomy^41^ additionally predicts S–S conflict during stimulus encoding in the Spatial Stroop task, which could have produced congruency effects in the target-aligned time series, no such effects were observed. Descriptively, the Spatial Stroop task showed a slightly more extended period of reduced moment variability in the response-aligned data (from 300 ms before response onset). Nevertheless, these observations indicate that despite the additional S–S conflict in the Spatial Stroop compared to the Simon task, the different dimensional overlap structure did not induce additional interference with balance control.

Both Simon and Spatial Stroop tasks demonstrated systematic modulation of force moment variability across the entire trial duration with a reduction in moment variability gradually emerging around target onset and the achievement of a relative minimum during response selection before onset of a response. This general dynamic might indicate tighter balance control when specific cognitive processing was required or at least expected. Koger et al.^57^ demonstrated similar general dynamics of moment variability across much longer trial periods in a setup comprising a cognitive dual-task. We believe that the global reduction of moment variability during a trial compared to the leading (and following) intertrial interval is an expression of strategically timed, proactive balance control in anticipation of impending cognitive demands. Thus, in the present study, we can hypothesize that two distinct components modulate moment variability. A global reduction during a cognitive trial irrespective of the congruency condition, and a local reduction during response selection, when conflict resolution is necessary for a correct response.

### Direction-specificity of interference effects on balance

In both our previous^34^ and the present study, the congruency effect on balance was substantially stronger in the ML direction. This directional specificity may relate to the spatial correspondence between the cognitive task’s response dimension (left–right button presses) and the ML plane of balance control, consistent with a task-specific, egocentric frame of reference.

It has been argued that the balance control system can independently adapt and respond to challenges in different directional planes through control mechanisms that decouple ML and AP balance control via direction-specific muscle synergies^62–64^. In addition to the evidence that body balance control in the mediolateral and anteroposterior planes can be dissociated, Scholz and Schöner^65^ suggested that the motor system strategically organizes movement variability into two subspaces due to motor system redundancy: it minimizes task-relevant variability that directly affects performance outcomes while it allows or even exploits task-redundant motor variability that does not compromise the task goal.

Transient suppression or reduction of variability in directions that threaten the stability of a task-relevant variable is selectively applied at critical task phases^66^. Possible mechanisms for stricter balance control by means of the reduction of variance could involve increased muscle co-contractions to stiffen joints, alterations in neural feedback control gains, or damping of exploratory behaviour, that are normally applied when boundary conditions are approached, constraints encountered, or critical events detected. Any interference observed between the cognitive task and balance control observed in these multitasking contexts has been debated as an indication of a facilitatory role of balance control or adaptive time-sharing of a central capacity^67–69^. In a multitasking situation, where some kind of precision behaviour in the suprapostural task is required, decomposition of joint variability into performance-relevant components and non-relevant components becomes more complex. In our present study, however, both cognitive tasks did not involve explicit precision demands, but minimized retinal slip at the time of target onset in the mediolateral direction might have been implicitly beneficial. In a follow-up experiment, we are currently testing if the direction-selective reduction in moment variability is dependent on an egocentric frame of reference that is imposed by the demands of the cognitive task.

### Interpretation of force moment variability

Force moment variability within a time bin indexes the degree of active corrective adjustments applied by the neuromuscular system. Its reduction is inherently ambiguous: it might reflect suppression or postponement of corrections (interference), a stiffening strategy that increases immediate stability at the cost of adaptive flexibility, or more efficient control requiring fewer corrections.

In the context of the present study, however, it is more appropriate to assume that we are constantly operating within a functional range of sway variability, rather than near a critical boundary threshold where balance failure is imminent. Within this functional range, reduced sway variability may be maladaptive rather than beneficial. Therefore, we interpret the congruency-related reduction in force moment variability as reflecting a temporary suppression or postponement of balance adjustments during cognitive conflict resolution, for several reasons. First, the effect is time-locked to the brief period of active conflict resolution and is absent during congruent trials, which makes a sustained strategic shift less likely, especially not on such a brief timescale. Winter et al.^70^ reported stiffness modulation during quiet standing in cycles of roughly 10 to 15 s periodicity. Second, the effect is specific to the mediolateral axis and to the response-aligned time window, consistent with a transient interference account rather than a global change in balance control strategy. Third, the direction of the effect (reduced activity during high cognitive demand) aligns with predictions from bottleneck models of cognitive-motor interference^33,69,71^. We acknowledge, however, that the current data cannot definitively distinguish between these interpretations. Future work employing perturbation paradigms, muscle co-contraction indices, or frequency-domain analyses would help clarify the functional significance of the observed reduction.

### Mechanisms of cognitive-balance interference

The pattern of findings is compatible with a “micro-bottleneck” account^34^ similar to the central bottleneck model^33^: cognitive processes for response selection or conflict monitoring and resolution^58^ cannot be deployed simultaneously to processes of balance control [see also^40^]. This account builds on models of intermittent, event-driven balance control^36,72^, which propose that corrections are initiated only when discrepancies between a predicted hold-state and the observed state exceed a threshold. If cognitive conflict resolution competes for the central resources involved in detecting or acting on these discrepancies, the initiation of balance corrections would be delayed during that period.

This perspective is consistent with the adaptive multiple-resource time-sharing theory^69,71^, which holds that the time scale for balance adjustments and for cognitive processing are mutually constraining. The notion that cognitive control engagement may inhibit, suppress, or delay activity of balance control can also be explained through neurocognitive mechanisms, such as inhibition and gating^73^. When cognitive control is strongly engaged by conflict monitoring or response inhibition^74^, the ongoing sensorimotor balance control loop may be actively suppressed or downregulated to prevent lower-level balance control activity from interfering or distracting higher level cognitive control. For instance, the transient reduction of the sensorimotor gain of corrective balance adjustments per se could have two parallel effects, such as reduced moment variability^75,76^ as well as less frequent bottom-up calls for high-level involvement. More specifically, when the balance system detects instability, sensorimotor networks may generate signals that demand attention. Over a short period of time, this strategy might not result in noticeable balance instability that would require high-level intervention like over longer durations. Therefore, central mechanisms may aim to suppress the intrusion of these sensorimotor signals to shield a cognitive task . Thus, when cognitive control is activated, the activation of competing sensorimotor representations may be downregulated or suppressed to facilitate the cognitive task set. A functional analogy may exist with sensory selection during balance control: just as balance control involves selecting informative sensory cues and suppressing misleading ones under challenging conditions^78–81^, cognitive control may similarly gate the flow of sensorimotor information during periods of highly conflicting and ambiguous input.

### Limitations

Several limitations should be noted. First, both cognitive conflict paradigms employed in this study involve spatial interference, with irrelevant location information conflicting with relevant spatial or non-spatial stimulus attributes. Our conclusions are therefore restricted to spatial conflict types, and it remains an open question whether the observed balance interference generalises to non-spatial cognitive conflicts. Testing paradigms such as a colour-word Stroop task (currently being investigated) or an Eriksen flanker task would be necessary to determine whether the effects reported here extend beyond spatially mediated conflict. Second, our primary balance analyses were restricted to trials following a congruent previous trial, which maximises sensitivity but also limits the proportion of data contributing to the key findings and the sensitivity analysis above addresses this concern. Finally, as discussed above, the interpretation of reduced force moment variability remains ambiguous, and future work employing complementary measures of balance control would help clarify the functional significance of the observed effects.

## Summary and conclusion

Our study investigated how two cognitive conflict paradigms, a Spatial Stroop task and a Simon task, interfere with balance control during upright standing using an event-related methodology. Our current observations successfully replicate and extend previous findings^34^ indicating that spatial response selection conflict can permeate into the domain of balance control. The two types of spatial cognitive conflict, S-R conflict in the Simon task and combined S-S/S-R conflict in the Spatial Stroop task, produce interference effects manifested as lower mediolateral force moment variability in incongruent compared to congruent trials during the response-preparation period. From a theoretical perspective, the results align with predictive models of balance control and theories of intermittent, event-driven balance adjustment systems, which suggest that discrete corrective actions are triggered only when stability estimates fall below critical thresholds. During periods in which higher cognitive demand is present and response selection processes are recruited for conflict resolution, a “micro-bottleneck” may result in balance control being adaptively constrained, for example by a temporary suppression or postponement of balance adjustments.

## Supplementary Information

Below is the link to the electronic supplementary material.

Supplementary Material 1

## Data Availability

All extracted data files are available from the figshare database (10.6084/m9.figshare.31305772).

## References

[1] Winter, D. A. Human balance and posture control during standing and walking. *Gait Posture* **3**(4), 193–214 (1995).

[2] Nashner, L. M. Adaptation of human movement to altered environments. *Trends Neurosci.* **5**, 358–361 (1982).

[3] Massion, J. Postural control system. *Curr. Opin. Neurobiol* **4**(6), 877–887 (1994). 7888772 10.1016/0959-4388(94)90137-6

[4] Ivanenko, Y. & Gurfinkel, V. S. Human postural control. *Front. Neurosci.* **12**, 171 (2018). 29615859 10.3389/fnins.2018.00171 PMC5869197

[5] Duarte, M. & Freitas, S. M. Revision of posturography based on force plate for balance evaluation. *Rev Bras Fisioter* **14**(3), 183–192 (2010). 20730361

[6] Winter, D.A., *Biomechanics and motor control of human movement*. Honoken, (NJ: John Wiley & Sons, 2005).

[7] Raymakers, J. A., Samson, M. M. & Verhaar, H. J. The assessment of body sway and the choice of the stability parameter(s). *Gait Posture* **21**(1), 48–58 (2005). 15536033 10.1016/j.gaitpost.2003.11.006

[8] Diamond, A. Executive functions. *Annu. Rev. Psychol.* **64**, 135–168 (2013). 23020641 10.1146/annurev-psych-113011-143750 PMC4084861

[9] Simon, J. R. & Rudell, A. P. Auditory S-R compatibility: the effect of an irrelevant cue on information processing. *J. Appl. Psychol.* **51**(3), 300–304 (1967). 6045637 10.1037/h0020586

[10] Stürmer, B. et al. Control over location-based response activation in the Simon task: behavioral and electrophysiological evidence. *J. Exp. Psychol. Hum. Percept. Perform.* **28**(6), 1345–1363 (2002). 12542132 10.1037//0096-1523.28.6.1345

[11] Fischer, R., Plessow, F. & Kiesel, A. Auditory warning signals affect mechanisms of response selection: Evidence from a Simon task. *Exp. Psychol.* **57**(2), 89–97 (2010). 20178922 10.1027/1618-3169/a000012

[12] Lu, C. H. & Proctor, R. W. The influence of irrelevant location information on performance: A review of the Simon and spatial Stroop effects. *Psychon. Bull Rev* **2**(2), 174–207 (1995). 24203654 10.3758/BF03210959

[13] Rosenbaum, D., Mama, Y. & Algom, D. Stand by Your Stroop: Standing up enhances selective attention and cognitive control. *Psychol. Sci.* **28**(12), 1864–1867 (2017). 28952883 10.1177/0956797617721270

[14] Busch, N. et al. Withstand control: standing posture differentially affects space-based and feature-based cognitive control through enhanced physiological arousal. *Sci. Rep.* **15**(1), 26347 (2025). 40685471 10.1038/s41598-025-11692-6 PMC12277459

[15] Smith, K. C. et al. Standing enhances cognitive control and alters visual search. *Atten. Percept. Psychophys.* **81**(7), 2320–2329 (2019). 31044397 10.3758/s13414-019-01723-6

[16] Straub, E. R. et al. Does body posture reduce the Stroop effect? Evidence from two conceptual replications and a meta-analysis. *Acta Psychol.* **224**, 103497 (2022). 10.1016/j.actpsy.2022.103497 35091208

[17] Caron, E. E. et al. Does posture Influence the Stroop effect?. *Psychol. Sci.* **31**(11), 1452–1460 (2020). 33017261 10.1177/0956797620953842

[18] Somen, M. M. et al. Does standing up enhance performance on the stroop task in healthy young adults? A systematic review and meta-analysis. *Int. J. Environ. Res. Public Health* **20**(3), 2319 (2023). 36767687 10.3390/ijerph20032319 PMC9915369

[19] Fenske, P., Bermeitinger, C. & Baess, P. No influence of sitting vs. standing on Simon effects. *Acta Psychol.* **263**, 106357 (2026). 10.1016/j.actpsy.2026.106357 41616473

[20] Salihu, A. T., Hill, K. D. & Jaberzadeh, S. Effect of cognitive task complexity on dual task postural stability: A systematic review and meta-analysis. *Exp. Brain Res.* **240**(3), 703–731 (2022). 35034175 10.1007/s00221-021-06299-y

[21] Stins, J. F. & Beek, P. J. A critical evaluation of the cognitive penetrability of posture. *Exp. Aging Res.* **38**(2), 208–219 (2012). 22404541 10.1080/0361073X.2012.660053

[22] Woollacott, M. & Shumway-Cook, A. Attention and the control of posture and gait: A review of an emerging area of research. *Gait. Posture* **16**(1), 1–14 (2002). 12127181 10.1016/s0966-6362(01)00156-4

[23] Blumle, A. et al. A cognitive intersensory interaction mechanism in human postural control. *Exp. Brain Res.* **173**(3), 357–363 (2006). 16491407 10.1007/s00221-006-0384-z

[24] Pitts, J., Kannan, L. & Bhatt, T. Cognitive task domain influences cognitive-motor interference during large-magnitude treadmill stance perturbations. *Sensors* **23**(18), 7746 (2023). 37765803 10.3390/s23187746 PMC10534402

[25] Van Humbeeck, N. et al. Multitasking across the lifespan in different task contexts. *Sci. Rep.* **14**(1), 11817 (2024). 38783047 10.1038/s41598-024-61859-w PMC11116417

[26] Wunderlich, A. et al. The impact of cognitive-motor interference on balance and gait in hearing-impaired older adults: A systematic review. *Eur. Rev. Aging Phys. Act.* **21**(1), 17 (2024). 38914940 10.1186/s11556-024-00350-x PMC11194914

[27] Kerr, B., Condon, S. M. & McDonald, L. A. Cognitive spatial processing and the regulation of posture. *J. Exp. Psychol. Hum. Percept. Perform* **11**(5), 617–622 (1985). 2932533 10.1037//0096-1523.11.5.617

[28] VanderVelde, T. J., Woollacott, M. H. & Shumway-Cook, A. Selective utilization of spatial working memory resources during stance posture. *NeuroReport* **16**(7), 773–777 (2005). 15858423 10.1097/00001756-200505120-00023

[29] Melzer, I., Benjuya, N. & Kaplanski, J. Age-related changes of postural control: effect of cognitive tasks. *Gerontology* **47**(4), 189–194 (2001). 11408723 10.1159/000052797

[30] Patterson, R.M., et al. Effect of age on performance of task with spatial conflict, In International conference on virtual rehabilitation (ICVR). IEEE: (Philadelphia, 2013) PA. p. 22-26.

[31] Barra, J. et al. Increasing cognitive load with increasing balance challenge: Recipe for catastrophe. *Exp. Brain Res.* **174**(4), 734–745 (2006). 16721607 10.1007/s00221-006-0519-2

[32] Koch, I. et al. Cognitive structure, flexibility, and plasticity in human multitasking-An integrative review of dual-task and task-switching research. *Psychol. Bull.* **144**(6), 557–583 (2018). 29517261 10.1037/bul0000144

[33] Pashler, H. Dual-task interference in simple tasks: Data and theory. *Psychol. Bull.* **116**(2), 220–244 (1994). 7972591 10.1037/0033-2909.116.2.220

[34] Johannsen, L. et al. Assessing the influence of cognitive response conflict on balance control: An event-related approach using response-aligned force-plate time series data. *Psychol. Res.* **87**(7), 2297–2315 (2023). 36862201 10.1007/s00426-023-01809-9 PMC10457244

[35] Mordkoff, J. T. & Gianaros, P. J. Detecting the onset of the lateralized readiness potential: A comparison of available methods and procedures. *Psychophysiology* **37**(3), 347–360 (2000). 10860412 PMC2898902

[36] Gawthrop, P. et al. Intermittent control models of human standing: Similarities and differences. *Biol. Cybern.* **108**(2), 159–168 (2014). 24500616 10.1007/s00422-014-0587-5 PMC3962584

[37] Gawthrop, P. et al. Intermittent control: A computational theory of human control. *Biol. Cybern.* **104**(1–2), 31–51 (2011). 21327829 10.1007/s00422-010-0416-4

[38] Loram, I. D., Gawthrop, P. J. & Lakie, M. The frequency of human, manual adjustments in balancing an inverted pendulum is constrained by intrinsic physiological factors. *J. Physiol.* **577**(Pt 1), 417–432 (2006). 16973712 10.1113/jphysiol.2006.118786 PMC2000665

[39] Lakie, M. & Loram, I. D. Manually controlled human balancing using visual, vestibular and proprioceptive senses involves a common, low frequency neural process. *J. Physiol.* **577**(Pt 1), 403–416 (2006). 16959857 10.1113/jphysiol.2006.116772 PMC2000668

[40] Muller, M. L. et al. Effect of preparation on dual-task performance in postural control. *J. Mot. Behav.* **36**(2), 137–146 (2004). 15130865 10.3200/JMBR.36.2.137-146

[41] Kornblum, S., Hasbroucq, T. & Osman, A. Dimensional overlap: cognitive basis for stimulus-response compatibility–a model and taxonomy. *Psychol. Rev.* **97**(2), 253–270 (1990). 2186425 10.1037/0033-295x.97.2.253

[42] Hommel, B. The Simon effect as tool and heuristic. *Acta Psychol (Amst)* **136**(2), 189–202 (2011). 20507830 10.1016/j.actpsy.2010.04.011

[43] Simon, J. R. Reactions toward the source of stimulation. *J. Exp. Psychol.* **81**(1), 174–176 (1969). 5812172 10.1037/h0027448

[44] Kornblum, S. Dimensional overlap and dimensional relevance in stimulus-response and stimulus-stimulus compatibility. In *Tutorials in motor behavior* (eds Stelmach, G. E. & Requin, J.) 743–777 (North-Holland, 1992).

[45] Li, Q. et al. Conflict detection and resolution rely on a combination of common and distinct cognitive control networks. *Neurosci. Biobehav. Rev.* **83**, 123–131 (2017). 29017916 10.1016/j.neubiorev.2017.09.032

[46] Hommel, B. Effects of irrelevant spatial S-R compatibility depend on stimulus complexity. *Psychol. Res.* **56**(3), 179–184 (1994). 8008780 10.1007/BF00419705

[47] Hommel, B. The role of attention for the Simon effect. *Psychol. Res.* **55**(3), 208–222 (1993). 8416040 10.1007/BF00419608

[48] Fischer, R. et al. Trial-to-trial modulations of the Simon effect in conditions of attentional limitations: Evidence from dual tasks. *J. Exp. Psychol. Hum. Percept. Perform.* **36**(6), 1576–1594 (2010). 20718574 10.1037/a0019326

[49] Faul, F. et al. G*Power 3: A flexible statistical power analysis program for the social, behavioral, and biomedical sciences. *Behav. Res. Method.* **39**(2), 175–191 (2007). 10.3758/bf03193146 17695343

[50] Hartmann, R. et al. forceplate: An R package for processing raw force-plate time-series data. *Behav. Res. Method.* **57**(7), 187 (2025). 10.3758/s13428-025-02657-8 PMC12133998 40461836

[51] Peirce, J. et al. PsychoPy2: Experiments in behavior made easy. *Behav. Res. Method.* **51**(1), 195–203 (2019). 10.3758/s13428-018-01193-y PMC6420413 30734206

[52] Liu, X. et al. Common and distinct neural substrates of attentional control in an integrated Simon and spatial Stroop task as assessed by event-related fMRI. *Neuroimage* **22**(3), 1097–1106 (2004). 15219581 10.1016/j.neuroimage.2004.02.033

[53] Gratton, G., Coles, M. G. & Donchin, E. Optimizing the use of information: Strategic control of activation of responses. *J. Exp. Psychol. Gen.* **121**(4), 480–506 (1992). 1431740 10.1037//0096-3445.121.4.480

[54] Moretti, L. et al. Quality over quantity: Focusing on high-conflict trials to improve the reliability and validity of attentional control measures. *J. Exp. Psychol. Learn. Mem. Cogn.* **52**(1), 34–54 (2026). 39946591 10.1037/xlm0001466

[55] Dienes, Z. Use one system for all results to avoid contradiction: Advice for using significance tests, equivalence tests, and Bayes factors. *J Exp Psychol Hum Percept Perform* **50**(5), 531–534 (2024). 38635164 10.1037/xhp0001202

[56] Hommel, B., Proctor, R. W. & Vu, K. P. A feature-integration account of sequential effects in the Simon task. *Psychol. Res.* **68**(1), 1–17 (2004). 14752663 10.1007/s00426-003-0132-y

[57] Koger, A. et al. Adjustments of balance control during cognitive dual tasking: Evidence from event-related force-plate analysis. *Psychol. Res.* **90**(1), 5 (2025). 41351648 10.1007/s00426-025-02215-z PMC12681466

[58] Botvinick, M. M. et al. Conflict monitoring and cognitive control. *Psychol Rev* **108**(3), 624–652 (2001). 11488380 10.1037/0033-295x.108.3.624

[59] Dignath, D. et al. Conflict monitoring and the affective-signaling hypothesis-An integrative review. *Psychon. Bull. Rev.* **27**(2), 193–216 (2020). 31898269 10.3758/s13423-019-01668-9

[60] Egner, T. Creatures of habit (and control): A multi-level learning perspective on the modulation of congruency effects. *Front. Psychol.* **5**, 1247 (2014). 25414679 10.3389/fpsyg.2014.01247 PMC4222221

[61] Fischer, R. et al. Individual differences in the context-dependent recruitment of cognitive control: Evidence from action versus state orientation. *J. Pers.* **83**(5), 575–583 (2015). 25297472 10.1111/jopy.12140

[62] Matjacić, Z. et al. Functional postural responses after perturbations in multiple directions in a standing man: A principle of decoupled control. *J. Biomech.* **34**(2), 187–196 (2001). 11165282 10.1016/s0021-9290(00)00182-2

[63] Winter, D. A. et al. Unified theory regarding A/P and M/L balance in quiet stance. *J. Neurophysiol.* **75**(6), 2334–2343 (1996). 8793746 10.1152/jn.1996.75.6.2334

[64] Gruneberg, C. et al. Spatio-temporal separation of roll and pitch balance-correcting commands in humans. *J. Neurophysiol.* **94**(5), 3143–3158 (2005). 16033938 10.1152/jn.00538.2004

[65] Scholz, J. P. & Schoner, G. The uncontrolled manifold concept: Identifying control variables for a functional task. *Exp. Brain Res.* **126**(3), 289–306 (1999). 10382616 10.1007/s002210050738

[66] Martin, V., Reimann, H. & Schoner, G. A process account of the uncontrolled manifold structure of joint space variance in pointing movements. *Biol. Cybern.* **113**(3), 293–307 (2019). 30771072 10.1007/s00422-019-00794-w PMC6510836

[67] Stoffregen, T. A. et al. Postural stabilization of looking. *J. Exp. Psychol. Human Percept. Perform.* **25**, 18 (1999).

[68] Mitra, S. Adaptive utilization of optical variables during postural and suprapostural dual-task performance: Comment on Stoffregen, Smart, Bardy, and Pagulayan (1999). *J. Exp. Psychol. Hum. Percept. Perform* **30**(1), 28–38 (2004). 14769066 10.1037/0096-1523.30.1.28

[69] Mitra, S. & Fraizer, E. V. Effects of explicit sway-minimization on postural–suprapostural dual-task performance. *Hum. Mov Sci* **23**(1), 1–20 (2004). 15201038 10.1016/j.humov.2004.03.003

[70] Winter, D. A. et al. Stiffness control of balance in quiet standing. *J. Neurophysiol.* **80**(3), 1211–1221 (1998). 9744933 10.1152/jn.1998.80.3.1211

[71] Fraizer, E. V. & Mitra, S. Postural costs of performing cognitive tasks in non-coincident reference frames. *Exp. Brain Res.* **185**(3), 429–441 (2008). 17960369 10.1007/s00221-007-1163-1

[72] Loram, I. D. et al. Is Intermittent control the source of the non-linear oscillatory component (0.2–2Hz) in human balance control?. *IEEE Trans. Biomed. Eng.* **69**(12), 3623–3634 (2022). 35560085 10.1109/TBME.2022.3174927

[73] Aron, A. R., Robbins, T. W. & Poldrack, R. A. Inhibition and the right inferior frontal cortex: One decade on. *Trends Cogn. Sci.* **18**(4), 177–185 (2014). 24440116 10.1016/j.tics.2013.12.003

[74] Swainson, R. et al. Cognitive control mechanisms revealed by ERP and fMRI: Evidence from repeated task-switching. *J. Cogn. Neurosci.* **15**(6), 785–799 (2003). 14511532 10.1162/089892903322370717

[75] Goodworth, A., Felmlee, D. & Karmali, F. Variation between individuals in sensorimotor feedback control of standing balance. *J. Neurophysiol.* **130**(2), 303–318 (2023). 37380599 10.1152/jn.00353.2022

[76] Le Mouel, C. Anticipatory coadaptation of ankle stiffness and sensorimotor gain for standing balance. *PLoS Comput. Biol.* **15**(11), e1007463 (2019). 31756199 10.1371/journal.pcbi.1007463 PMC6897426

[77] Goschke, T. & Dreisbach, G. Conflict-triggered goal shielding: response conflicts attenuate background monitoring for prospective memory cues. *Psychol. Sci.* **19**(1), 25–32 (2008). 18181788 10.1111/j.1467-9280.2008.02042.x

[78] Haggerty, S. E. et al. A shared neural integrator for human posture control. *J. Neurophysiol.* **118**(2), 894–903 (2017). 28446583 10.1152/jn.00428.2016 PMC5539436

[79] Hwang, S. et al. Dynamic reweighting of three modalities for sensor fusion. *PLoS ONE* **9**(1), e88132 (2014). 24498252 10.1371/journal.pone.0088132 PMC3909337

[80] Redfern, M. S. et al. Attention influences sensory integration for postural control in older adults. *Gait. Posture* **14**(3), 211–216 (2001). 11600324 10.1016/s0966-6362(01)00144-8

[81] Redfern, M. S. et al. Perceptual inhibition is associated with sensory integration in standing postural control among older adults. *J. Gerontol. B Psychol. Sci. Soc. Sci.* **64**(5), 569–576 (2009). 19617457 10.1093/geronb/gbp060 PMC2800814

