# Supplementary material for: Contrasting cognitive control in the Simon and spatial Stroop tasks regarding their interference with the control of standing balance
Source: Sci Rep. 2026 Jun 9;16:17838. doi: 10.1038/s41598-026-56904-9 (PMC13249827; doi:10.1038/s41598-026-56904-9)
Supplement: Supplementary file 1 — Supplementary Material 1 [file 41598_2026_56904_MOESM1_ESM.docx]

**Supplementary materials: Contrasting cognitive control in the Simon and spatial Stroop tasks regarding their interference with the control of standing balance**

Leif Johannsen^1^, Anton Koger^1^, Elisa Ruth Straub^2^, Denise Nadine Stephan^1^, Andrea Kiesel^2^, Iring Koch^1^, Hermann Müller^3^

^1^ Institute of Psychology, RWTH Aachen University, Germany

^2^ Department of Psychology, University of Freiburg, Germany

^3^ Department of Sport Science, University of Giessen, Germany

Address for correspondence

Leif Johannsen, PD Dr rer nat habil, Dipl-Psych, MSc

Cognitive and Experimental Psychology

Institute of Psychology

RWTH Aachen University, Germany

Address: Jaegerstr. 17/19, D-52066 Aachen, Germany

Tel.: +49 241 80 96 488

Email: Leif.Johannsen@psych.rwth-aachen.de

ORCID: 0000-0002-2441-3163

**Supplementary results**

**Analysis of Manual Reactions**

As balance control was analysed separately for each cognitive task condition, we also analysed the cognitive tasks in a combined analysis but also in two separate analyses.

**Comparison Between Both Cognitive Tasks**

An ANOVA on RT including both cognitive tasks (Simon task and Spatial Stroop task) indicated that RT were shorter in the Spatial Stroop task (M=490 ms, SD=60) than the Simon task (M=501 ms, SD=68; F(1, 89)=4.58, MSE=4503.04, p=.04, ƞ_p_^2^=.05). On congruent trials, RTs (M=480 ms, SD=63) were shorter than incongruent RTs (M=511 ms, SD=61; F(1, 89)=286.98, MSE=606.84, p<.001, ƞ_p_^2^=.76). Further, a main effect of previous trial congruency was observed (F(1, 89)=60.79, MSE=108.35, p<.001, ƞ_p_^2^=.41) with faster responses following a previously congruent trial (M=492 ms, SD=67) than an incongruent trial (M=498, SD=60) , which, however, was qualified by an interaction between previous trial congruency and current trial congruency (F(1, 89)=790.18, MSE=324.80, p<.001, ƞ_p_^2^=.90). When the previous trial was congruent, then RTs in congruent trials were shorter than in incongruent trials, resulting in a congruency effect of 69 ms, t(89) =30.23, p<0.001, dz=3.19, but when the previous trial was incongruent, then RTs in congruent trials were longer relative to RT on incongruent trials, resulting in a “reversed” congruency effect of 7 ms (t(89)=2.93, p=0.004, dz=0.31). We also observed an interaction between cognitive task and congruency (F(1, 89)= 20.65, MSE=260.74, p<.001, ƞ_p_^2^=.19), which indicated a greater overall congruency effect in the Spatial Stroop task (M=36 ms; t(89)=14.64, p<0.001, dz=1.54) compared to the Simon task (M=26 ms; t(89)=13.90, p<0.001, dz=1.47).

Finally, a three-way interaction between cognitive task, congruency and previous-trial congruency indicated, that the congruency sequence effect described above differed between the two cognitive tasks (F(1, 89)=10.95, MSE=112.54, p=.001, ƞ_p_^2^=.11).

Both the Simon task and the Spatial Stroop demonstrated individual main effects of congruency and previous congruency as well as an interaction between both factors (statistic effects for each task are reported in the supplementary materials and Table 4). In the Simon task, when the previous trial was congruent, then a Simon effect of 61 ms was observed (t(89)=25.97, p<0.001, dz=2.74) but when the previous trial was incongruent, then a “reversed” Simon effect of 10 ms (incongruent trials resulted in shorter RTs than congruent trials) occurred (t(89)=3.53, p=0.0007, dz=0.37). Figure 2a shows the reaction times for the Simon task. In the Spatial Stroop task, when the previous trial was congruent, then a Spatial Stroop effect of 77 ms was observed (t(89)=25.78, p<0.001, dz=2.72) but, in contrast to the Simon task, when the previous trial was incongruent, then no difference was observed between congruent and incongruent trials (M=-3 ms; t(89)=1.40, p=0.16, dz=0.15). Figure 3a shows the reaction times for the Spatial Stroop task.

The error proportions demonstrated lower error proportions in the Spatial Stroop task (M=3.0 %, SD=3.8) compared to the Simon task (M=3.6 %, SD=4.1; F(1, 89)=9.84, MSE=7.05, p=.002, ƞ_p_^2^=.10). Additionally, we observed an effect of congruency (F(1, 89)=128.42, MSE=11.27, p<.001, ƞ_p_^2^=.59) with more errors in incongruent trials (M=4.7%, SD=4.5) compared to congruent trials (M=1.9%, SD=2.6) and an effect of previous trial congruency (F(1, 89)=103.71, MSE=3.31, p<.001, ƞ_p_^2^=.54) with higher error proportions (M=4.0%, SD=4.8) when the previous trial was congruent in contrast to when it was incongruent (M=2.6%, SD=2.7). An interaction between previous trial congruency and congruency was also found (F(1, 89)=197.46, MSE=10.73, p<.001, ƞ_p_^2^=.69). When the previous trial was congruent, then error proportions in incongruent trials were higher than in congruent trials, resulting in a congruency effect of 6.3% (t(89)=14.1, p<0.001, dz=1.49), but when the previous trial was incongruent, then error proportions in incongruent trials were even lower than in congruent trials, resulting in a “reversed” congruency effect of 0.6% (t(89)=2.74, p=0.007, dz=0.29), mirroring the RT data. An interaction between cognitive task and congruency (F(1, 89)=12.92, MSE=3.72, p<.001, ƞ_p_^2^=.13) indicated that in the Spatial Stroop task the congruency effect was greater (M=3.4 %; t(89)=12.20, p<0.001, dz=1.29) compared to the Simon task (M=2.1 %; t(89)=7.68, p<0.001, dz=0.81). The interaction between cognitive task and previous trial congruency was also significant (F(1, 89) = 7.50, MSE=2.15, p = .007, ƞ_p_^2^ = .08).

Again, like in the RT data, a three-way interaction between cognitive task, congruency and previous-trial congruency was also found. It indicated differences in the congruency sequence effect between the two cognitive tasks (F(1, 89)=4.34, MSE=2.01, p=.04, ƞ_p_^2^=.05). In the Simon task, when the previous trial was congruent, then error proportions in congruent trials were lower than in incongruent trials, resulting in a Simon effect of 5.9% (t(89)=12.12, p<0.001, dz=1.28) but when the previous trial was incongruent, then, consistent with the RT data, error proportions on congruent trials were even higher than on incongruent trials, resulting in a “reversed” Simon effect of 1.3% (t(89)=4.23, p<0.001, dz=0.45). Figure 2b shows the error proportions for the Simon task. In the Spatial Stroop task, when the previous trial was congruent, then error proportions in congruent trials were lower than in incongruent trials, resulting in a Spatial Stroop effect of 6.5% (t(89)=13.81, p<0.001, dz=1.46), but when the previous trial was incongruent, like for the RTs, then error proportions did not differ between congruent and incongruent trials (M=0.1%; t(89)=0.65, p=0.52, dz=0.07). Figure 3b shows the error proportions for the Spatial Stroop task.

**Manual Simon Task**

For the Simon task, a two-way ANOVA revealed shorter RTs on congruent trials than on incongruent trials (F(1, 89)=193.28, MSE=306.01, p<.001, ƞ_p_^2^=.68). The main effect of previous-trial congruency was also significant (F(1, 89)=17.62, MSE=127.93, p<.001, ƞ_p_^2^=.17) and there was an interaction between previous trial congruency and congruency (F(1, 89)=414.90, MSE=267.92, p<.001, ƞ_p_^2^=.82).

For the error proportions, the ANOVA revealed a main effect of congruency (F(1, 89)=59.05, MSE=8.19, p<.001, ƞ_p_^2^=.40) showing higher error rates for incongruent trials than for congruent trials (4.8% vs. 2.5%) and thus a Simon effect of 2.3%. The main effect of previous-trial congruency (F(1, 89)=37.61, MSE=2.80, p<.001, ƞ_p_^2^=.30) was significant. That is, when the previous trial was congruent, error proportions were higher (4.2%) than when the previous trial was incongruent (3.1%), which expresses generally improved performance after experiencing response selection conflict. Importantly though, the interaction between congruency and previous trial congruency was significant (F(1, 89)=166.97, MSE=7.18, p<.001, ƞ_p_^2^=.65).

**Manual Spatial Stroop Task**

For the Spatial Stroop task, a similar two-way ANOVA revealed shorter RTs on congruent trials than on incongruent trials (F(1, 89)=214.39, MSE=561.57, p<.001, ƞ_p_^2^=.71). The main effect of previous-trial congruency was also significant (F(1, 89)=57.00, MSE=79.46, p<.001, ƞ_p_^2^=.39) and there was an interaction between previous trial congruency and congruency (F(1, 89)=866.01, MSE=169.42, p<.001, ƞ_p_^2^=.91).

For the error proportions, the ANOVA revealed a main effect of congruency (F(1, 89)=148.79, MSE=6.80, p<.001, ƞ_p_^2^=.63) showing higher error rates for incongruent trials than for congruent trials (4.7% vs. 1.3%) and thus a Spatial Stroop effect of 3.4%. The main effect of previous-trial congruency (F(1, 89)=95.57, MSE=2.65, p<.001, ƞ_p_^2^=.52) was significant. That is, when the previous trial was congruent, error proportions were higher (3.8%) than when the previous trial was incongruent (2.2%), which expresses generally improved performance after experiencing response selection conflict. Importantly, the interaction was significant (F(1, 89)=167.13, MSE=5.55, p<.001, ƞ_p_^2^=.65).

**Analysis Of Force Moment Variability**

**Target‑Aligned And Response‑Aligned Time Series Analyses Of Congruency Effects In The Simon Task**

For the target-aligned variability of force moment in the Simon task in the anteroposterior direction, a modulation across the six time bins from 150 ms before to 300 ms after target onset was observed (F(2.81, 249.75)= 34.62, MSE=0.0074, p<.001, ƞ_p_^2^=.28; Fig. S1a), which amounted to a gradual reduction in moment variability beginning in the time bins from 75 ms after target onset. In the mediolateral direction, moment variability was also altered across the six time bins (F(3.16, 281.32)= 63.69, MSE=0.0066, p<.001, ƞ_p_^2^=.42; Fig. 5c). A gradual reduction in moment variability began in the time bins from 75 ms after target onset. An interaction between congruency and time bins remained marginally significant only (F(3.33, 296.76) = 2.31, MSE=0.0054, p = .070, ƞ_p_^2^ = .03). Following up this marginal interaction nevertheless indicated that for the mediolateral moment variability, a significant effect of congruency was observed in the two time bins from 75 ms before to 75 ms after target onset only (both F(1,89) > 5.31, both p<0.024, both ƞ_p_^2^ > 0.06; Fig. S1c). In both time bins, the moment variability was less in incongruent trials (M_1_=-2.60, SD_1_=0.47; M_2_=-2.63, SD_2_=0.47) compared to congruent trials (M_1_=-2.57, SD_1_=0.57; M_2_=-2.60, SD_2_=0.57).

Please note that congruency should not result in differences in the time bin 75 ms before target onset until target onset, because participants cannot know whether the target will be congruent or incongruent in this time bin. Thus, for evaluating if the congruency effect observed in the time bin from 75 ms before target onset until target onset was caused by potential inaccuracies in the timing of the event triggers signalling target onset, we conducted additional ANOVAs for a set of time bins that were shifted by 25 ms to the left resulting in five time bin ranging from 100 ms before to 275 ms after target onset. In this analysis, the time bin 100 ms to 25 ms before target onset did not differ depending on congruency. Only the time bin that overlapped with the target onset (from 25 ms before to 50 ms after) showed reduced force moment variability in incongruent trials (M=-2.62, SD=0.50) compared to congruent trials (M=-2.59, SD=0.52; F(1, 89)=6.21, MSE=0.0076, p=.015, ƞ_p_^2^=.07; Tables S1, S2 and Figure S2b). The preceding time bin did show a marginal effect of congruency still (F(1, 89) = 3.91, MSE=0.0059, p = .051, ƞ_p_^2^ = .04). It is a remarkable observation that force moment variability in the bin from 75 ms before to target-onset was reduced in incongruent trials in the target-aligned time series data too. As all trials analyzed here were post-congruent, it may also be the case that this phenomenon as an indication of pre-trial anticipation of a required switch to an incoming incongruent target.

Shifting the bin ranges by 25 ms to the left (supplementary Table S1 and Figure S1) abolished this effect and left an effect of congruency around the time of the onset of a target. Currently, we are unsure how to interpret this early effect of congruency on balance control. The effect may be an expression of preparatory engagement that ceases when the target is finally (the total ITI could last up to 2.7 s in duration) revealed as congruent. As this phenomenon did not occur in the Spatial Stroop task, it is possible that it represents a false positive finding.

For the response-aligned variability of force moment in the Simon task in the anteroposterior direction, a modulation of moment variability across the six temporal bins from 300 ms before to 150 ms after response onset was observed (F(2.64, 234.87)=8.05, MSE=0.0096, p<.001, ƞ_p_^2^=.08; Fig. S1b). Moment variability was smallest in the two time bins before response onset with a gradual increase from the response onwards. In the mediolateral direction, moment variability was also altered across the six time bins (F(2.40, 213.82)=8.73, MSE=0.011, p<.001, ƞ_p_^2^=.09; Fig. S1d). Moment variability again was smallest in the two time bins before response onset with a gradual increase from the response onwards.

In the anteroposterior direction, for the response-aligned time bins, no significant differences in any time bins that showed an effect of congruency were found. In contrast in the mediolateral direction, the Simon task demonstrated temporal bins with significant effects of congruency before response onset (F(1, 89) = 5.08, MSE= 0.0295, p = .027, ƞ_p_^2^ = .05). The three time bins from 225 ms before to response onset demonstrated significant effects of congruency (all F(1,89) > 5.00, all MSE< 0.0065, all p<0.028, all ƞ_p_^2^ > 0.05; Fig. S1d). The single bin with the greatest effect size was the time bin from 150 ms to 75 ms before response onset. Moment variability was lower in incongruent (M_2_=-2.70, SD_2_=0.48; M_3_=-2.73, SD_23_=0.48; M_4_=-2.73, SD_4_=0.45) compared to congruent trials (M_2_=-2.67, SD_2_=0.50; M_3_=-2.69, SD_3_=0.50; M_4_=-2.70, SD_4_=0.48).

--- Tables S1 and S2 and Figures S1 and S2 about here ---

**Target‑Aligned And Response‑Aligned Time Series Analyses Of Local Congruency Effects In The Spatial Stroop Task**

For the target-aligned variability of force moment in the Spatial Stroop task in the anteroposterior direction, a modulation across the six temporal bins from 150 ms before to 300 ms after target onset was observed (F(3.65, 324.90)=48.05, MSE=0.0040, p<.001, ƞ_p_^2^=.35; Fig. S3a). A gradual reduction in moment variability began in the time bins from 75 ms after target onset. In the mediolateral direction, moment variability was also altered across the six time points (F(3.52, 312.88)= 60.15, MSE=0.0050, p<.001, ƞ_p_^2^=.40; Fig. S3c). Moment variability showed its minimum in the two time bins before response onset with a gradual increase from the response onwards. In the Spatial Stroop task, target-alignment did not result in any time bins with significant congruency effects in either direction.

For the response-aligned variability of force moment in the Spatial Stroop task in the anteroposterior direction, a modulation across the six temporal bins from 300 ms before to 150 ms after response onset was observed (F(2.71, 240.94)=9.46, MSE=0.0087, p<.001, ƞ_p_^2^=.10; Fig. S3c). A gradual reduction in moment variability until the time bin from 75 ms before to response onset occurred. In the mediolateral direction, moment variability was also altered across the six time points (F(2.46, 219.32)=11.47, MSE=0.010, p<.001, ƞ_p_^2^=.11; Fig. S3d). Moment variability was smallest in the two time bins before response onset with a gradual increase from the response onwards.

Similar to the Simon task, no significant differences depending on congruency were found in time bins in the anteroposterior direction in the Spatial Stroop task. In the mediolateral direction, a main effect of congruency was significant (F(1, 89) = 7.63, MSE= 0.0244, p = .007, ƞ_p_^2^ = .08) and four time bins that ranged from 300 ms before to response onset differed depending on congruency. The largest effect size was observed in the time bin from 75 ms to response onset (all F(1,89) > 4.72, all MSE< 0.0090, all p<0.032, all ƞ_p_^2^ > 0.05; Fig. S3d). Again, moment variability was lower in incongruent trials (M_1_=-2.67, SD_1_=0.53; M_2_=-2.69, SD_2_=0.53; M_3_=-2.72, SD_3_=0.52; M_4_=-2.72, SD_4_=0.51) compared to congruent trials (M_1_=-2.64, SD_1_=0.53; M_2_=-2.66, SD_2_=0.55; M_3_=-2.68, SD_3_=0.53; M_4_=-2.68, SD_4_=0.53).

--- Figure S3 about here ---

Tables

| Cognitive conflict task | Time bin | -100 to -25 ms | | -25 to 50 ms | | 50 to 125 ms | | 125 to 200 ms | | 200 to 275 ms | |
| --- | --- | --- | --- | --- | --- | --- | --- | --- | --- | --- | --- |
|  |  | M | SD | M | SD | M | SD | M | SD | M | SD |
|  | Congruency |  |  |  |  |  |  |  |  |  |  |
| Simon task | Incongruent | -2.60 | 0.50 | -2.62 | 0.50 | -2.63 | 0.49 | -2.65 | 0.51 | -2.68 | 0.49 |
|  | Congruent | -2.58 | 0.53 | -2.59 | 0.52 | -2.62 | 0.53 | -2.64 | 0.51 | -2.67 | 0.50 |
|  | Congruency effect | -0.023 | 0.11 | -0.032 | 0.12 | -0.012 | 0.13 | -0.011 | 0.13 | -0.005 | 0.11 |
| Spatial Stroop  task | Incongruent | -2.59 | 0.56 | -2.60 | 0.56 | -2.61 | 0.55 | -2.63 | 0.55 | -2.66 | 0.55 |
|  | Congruent | -2.58 | 0.56 | -2.59 | 0.55 | -2.61 | 0.55 | -2.63 | 0.55 | -2.66 | 0.53 |
|  | Congruency effect | -0.008 | 0.12 | -0.009 | 0.11 | 0.003 | 0.11 | 0.002 | 0.09 | -0.004 | 0.10 |

Table S1. Descriptive statistics of the target-aligned mediolateral force moment variability for both cognitive tasks as a function of congruency and time bin (shifted by -25 ms). M: mean, S: standard deviation.

|  | |  | F, p, partial eta^2 | | | | |
| --- | --- | --- | --- | --- | --- | --- | --- |
|  |  | Temporal bin | -100 to -25 ms | -25 to 50 ms | 50 to 125 ms | 125 to 200 ms | 200 to 275 ms |
| Target-aligned | Simon task | Anteroposterior | F(1, 89)= 0.15, p=.70, **ƞ_p_^2^**<.01 | F(1, 89)= 0.05, p=.82, **ƞ_p_^2^**<.01 | F(1, 89)= 0.87, p=.35, **ƞ_p_^2^**<.01 | F(1, 89)= 0.25, p=.62, **ƞ_p_^2^**<.01 | F(1, 89)= 0.12, p=.73, **ƞ_p_^2^**<.01 |
|  |  | Mediolateral | F(1, 89)= 3.91, p=.05, **ƞ_p_^2^**=.04 | **F(1, 89)= 6.21, p=.02, ƞ_p_^2^=.07** | F(1, 89)= 0.79, p=.38, **ƞ_p_^2^**<.01 | F(1, 89)= 0.64, p=.43, **ƞ_p_^2^**<.01 | F(1, 89)= 0.16, p=.69, **ƞ_p_^2^**<.01 |
|  | Spatial Stroop task | Anteroposterior | F(1, 89)= 1.49, p=.23, **ƞ_p_^2^**=.02 | F(1, 89)= 0.04, p=.84, **ƞ_p_^2^**<.01 | F(1, 89)= 1.25, p=.27, **ƞ_p_^2^**=.01 | F(1, 89)= 0.72, p=.40, **ƞ_p_^2^**<.01 | F(1, 89)= 0.37, p=.55, **ƞ_p_^2^**<.01 |
|  |  | Mediolateral | F(1, 89)= 0.51, p=.48, **ƞ_p_^2^**<.01 | F(1, 89)= 0.62, p=.43, **ƞ_p_^2^**<.01 | F(1, 89)= 0.06, p=.81, **ƞ_p_^2^**<.01 | F(1, 89)= 0.05, p=.82, **ƞ_p_^2^**<.01 | F(1, 89)= 0.19, p=.67, **ƞ_p_^2^**<.01 |

Table S2. Test statistics of ANOVAs of the congruency effect for the moment variability in each temporal bin (target-aligned) for both task conditions and both directions of body sway. Significant effects or interactions are indicated in bold.

Figures

Figure S1. Raincloud plots of the distributions of the anteroposterior (A) and mediolateral (B) log-transformed standard-deviation of moment variability in the Simon task across the target-aligned temporal bins as a function of congruency (only for trials where the previous trial was congruent too) for each extracted time bin (75 ms width; from 150 ms before to 300 ms after target onset). Raincloud plots of the distributions of the log-transformed standard-deviation of moment variability across the response-aligned temporal bins in the anteroposterior (C) and mediolateral (D: from 300 ms before to 150 ms after response onset). Significant effects are indicated with asterisks (*: p<0.05, **: p<0.01).

Figure S2. Raincloud plots of the distributions of the anteroposterior (A) and mediolateral (B) log-transformed standard-deviation of moment variability in the Simon task across the target-aligned temporal bins as a function of congruency (only for trials where the previous trial was congruent too) for each extracted time bin (75 ms width; from 100 ms before to 275 ms after target onset). Raincloud plots of the distributions of the log-transformed standard-deviation of moment variability across the target-aligned temporal bins in the anteroposterior (C) and mediolateral (D) direction in the Spatial Stroop task (from 100 ms before to 275 ms after target onset). Significant effects are indicated with asterisks (*: p<0.05).

Figure S3. Raincloud plots of the distributions of the anteroposterior (A) and mediolateral (B) log-transformed standard-deviation of moment variability in the Spatial Stroop task across the target-aligned temporal bins as a function of congruency (only for trials where the previous trial was congruent too) for each extracted time bin (75 ms width; from 150 ms before to 300 ms after target onset). Raincloud plots of the distributions of the log-transformed standard-deviation of moment variability across the response-aligned temporal bins in the anteroposterior (C) and mediolateral (D: from 300 ms before to 150 ms after response onset). Significant effects are indicated with asterisks (*: p<0.05, **: p<0.01; ***: p<0.001).
